# Considering similarity and the rating conversion of neighbors on neural collaborative filtering

**DOI:** 10.1371/journal.pone.0266512

**Published:** 2022-05-05

**Authors:** Thitiporn Neammanee, Saranya Maneeroj, Atsuhiro Takasu

**Affiliations:** 1 Department of Mathematics and Computer Science, Faculty of Science, Chulalongkorn University, Bangkok, Thailand; 2 Advanced Virtual and Intelligent Computing Center, Department of Mathematics and Computer Science, Faculty of Science, Chulalongkorn University, Bangkok, Thailand; 3 National Institute of Informatics The Graduate University for Advanced Studies, SOKENDAI, Tokyo, Japan; Education University of Hong Kong, CHINA

## Abstract

One of the most popular recommender system techniques is collaborative filtering (CF). Nowadays, many researchers apply a neural network with CF, but few focus on the neighbors’ concept of CF. This work needs to consider two major issues: the similarity levels between the neighbors and the target user and the user’s rating pattern conversion. Because different neighbors have a different influence on the target user and different users usually have a different rating pattern, the ratings directly utilized by the neighbor’s preference pattern may be incorrect for the target user. Under two main issues, we try to accomplish three main ideas of CF’s prediction: the similarity between users’ levels, the neighbor’s rating, and the rating conversion. Thus, we propose three main modules, the rating conversion module, the similarity module, and the prediction module, to solve the two issues mentioned above. The rating conversion module projects the neighbor’s rating into the target user’s aspect. The similarity module uses the users’ attentions to compute similarity levels between users. Finally, these similarity levels and the converted ratings are integrated to perform the prediction. The proposed method is compared with the current CF with friends and latent factor model using two types of datasets: real-world and synthetic datasets. We evaluate *N* neighbors and all neighbors on real-world datasets to prove the number of neighbor is important. Moreover, the performance of the rating conversion module is also evaluated. The proposed method simulates the full rating datasets and the partial rating dataset to compare the effectiveness of using different types of distribution and dataset size. The experimental results demonstrate that the proposed method effectively outperformed the baselines using ranking evaluation and prediction accuracy on real-world and synthetic datasets. Besides, The effectiveness of using different the number of neighbors depends on the quality of neighbors.

## 1 Introduction

A recommender system (RS) [1, 2] is an engine that helps predict the rating or preference for recommending items to the target user based on the user’s profile. Generally, RS process has three steps: preprocessing data, finding neighbors who have a similar preference to the target user, and recommending items preferred by neighbors to the target user. The three most popular techniques to develop a RS are content-based filtering (CBF) [3], collaborative filtering (CF) [4], and hybrid RS (HRS) [5]. HRS integrates both CF and CBF. The CBF approach recommends new items based on the content of the past items. For example, user A liked Harry Potter movies, which is the fantasy movie genre. A fantasy movie that has similar content to Harry Potter will then be recommended to the user A. The CF approach uses the neighbor’s preference for items recommendation to the target user. These recommenced items can be the items in the same or different categories. It means the CF technique can provide cross domain recommendation. While using the CBF will suggest items that are similar to the items the target user has rated. Suppose user A likes fantasy movies, and user B likes action movies in addition to fantasy. If B is a neighbor of A, the action movie that B likes will be recommended to A. Due to using the preference of the neighbors and the target user are enough to recommend the items. Therefore, it does not require content analysis or content extraction. In summary, the CF is the traditional idea and the most practical because using the neighbors to help in the prediction. It can be applied with other technical idea to improve the recommendation accuracy.

The matrix factorization (MF) [6, 7] technique, which was proposed in 2009 by Koren et al. [8], is the most popular model for RS. It decomposes a matrix into a lower dimension, which is used to compute the predicted rating of the target user on the target item. In the CF method, the rating matrix is decomposed into latent feature vectors of user and item, representing the user profiles and the item characteristics, respectively. Moreover, the MF technique can be applied to latent feature extraction methods such as principal component analysis (PCA) [9], singular value decomposition (SVD) [10], and latent Dirichlet allocation (LDA) [11]. When applying the MF technique to RS, user–item interactions are modeled as an inner product in the latent factor space. The equation of the MF concept is shown in (1):

$$\begin{array}{c} r_{ui}=p_{u}^{T}\cdot q_{i}. \end{array}$$ (1)

where *r*_*ui*_ denotes the estimate value of user *u*’s rating on item *i*, *p*_*u*_ is the latent factor of user *u*, and *q*_*i*_ is the latent factor of item *i*.

The example of applying the MF technique is the local geographical based logistic MF model (LGLMF) for point of view (POI) recommendation [12], which is used to detect the probability of user’s interest in the POI. Their work factorizes the check-in interaction of users and the POI matrix using logistic MF to obtain the probability that the target user has a preference toward the POI. A Posterior-neighborhood-regularized Latent Factor Model for Highly Accurate Web Service QoS Prediction (PLF) [13] utilizes the MF technique to extract and improve a user’s latent factor and item’s latent factor from a rating matrix by integrating similarity values and neighbors into these latent factors.

Currently, a neural network (NN) is widely used in word embedding and machine learning, such as recurrence NN (RNN) [14], convolutional NN (CNN) [15], and long short-term memory (LSTM) [16–18]. Applying a NN to the models makes the models more accurate and flexible. A basic NN comprises three components: the input layer, hidden layer, and output layer. Each layer consists of many nodes or neurons. All of the input variables are represented as input nodes in the input layer. Then, all of the input nodes are computed across one or more nodes in the hidden layer. Finally, the outputs from the hidden layer nodes are used to predict or classify user’s preference. A Clustering-Based Collaborative Filtering Recommendation Algorithm via Deep Learning User Side Information (AK-UCF) [19] presented a combination of auto-encoding network and an old fashion of CF technique, a clustering approach, to perform a recommendation list. Unfortunately, their work could not incorporate a neural network and the CF technique into one model.

A NN is also used in the RS area, such as neural CF (NCF) [20], the Outer Product-based Neural Collaborative Filtering (ONCF) [21], and temporal CNN for reviews based on recommender system (TCR) [22]. In NCF, generalized MF (GMF) and multilayer perceptron (MLP) NN models are combined to added the information into the users’ ratings. ONCF aim to improve the NCF using CNN instead of MLP, whereas in TCR, a time component is added into the CNN model, which helps to adjust users’ importance in chronological order. However, NCF, ONCF, and TCR only learn user–item interaction on rating. These models do not concern how much each user affects the target user as in the neighbor concept in the CF technique.

Recently, some researchers tried to apply the neighbors’ concept of the CF technique into their NN model such as A Neural Collaborative Filtering Model with Interaction-based Neighborhood (NNCF) [23], Recommendation Based on BP Neural Network with Attention Mechanism(BPAM) [24], the collaborative memory network for recommendation systems (CMN) [25] and the social attentional memory network: modeling aspect and friend-level differences in recommendation (SAMN) [26].

To build a CF model leveraging the neighbor’s concept, there are two keys need to concern, which are the similarity between the neighbor and the target user, the neighbor’s raiting. The NNCF utilized neighbors explicitly in their model, but it did not consider the importance level of neighbors as in the CF concept. The BPAM model proposed to use an impact of the target user on the neighbor, which creates a similarity between the neighbor and the target user. However, it did not compute or apply the neighbors’ ratings that need to be concerned in CF’s technique.

CMN and SAMN create a target user’s profile that combines all neighbor’s influences. Afterward, the target user’s profiles are merged with the target item to perform a prediction. The neighbor’s influences are the outcome of the similarity between the neighbors and the target user and the neighbor’s embedding combination. Both current NCFs with friends use the neighbor’s embedding and the target user’s embedding to compute similarity between the neighbor and the target user. The significant difference between CMN and SAMN is in the prediction process. After the neighbor’s influence is obtained, CMN uses the neighbor’s influence to learn with the target user embedding and the target item embedding through a NN. In comparison, SAMN combines the neighbor’s influence with the target user embedding to create the target user’s profile. Then, the target user’s profile is merged with the item embedding using a dot product to predict the rating as in the MF concept. Both current NCFs with friends still cannot simulate the CF technique prediction process. The CF’s prediction equation is a weighted average on the neighbors’ ratings where the similarities between the neighbors and the target user are used as a weight. However, both current NCFs with friends do not directly use the predicted rating from the neighbor’s rating. That is, no process in both methods generates the neighbor’s ratings toward the target item. These neighbor’s ratings are combined with the similarities between the neighbors and the target user to obtain the predicted rating as the CF’s rating prediction equation.

From two keys of CF’s technique, there are another issue in the CF technique is that different users have different rating ranges. Many CF application works usually use neighbor’s ratings to predict the target user rating without converting the rating range into the target user aspect. Both current NCFs with friends do not apply the neighbor’s ratings in the prediction process. Thus, both current NCFs with friends still do not convert the neighbor’s rating range into the target user aspect, thus leading to the rating conversion issue and making the prediction inaccurate.

There are two issues need to be considered: the rating conversion and the similarity among users. Under these two main issues, we desire to accomplish three main ideas of CF’s prediction: the similarity between users’ levels, the neighbor’s rating, and the rating conversion. First, create a similarity between the neighbor and the target user, Applying the neighbors’ ratings into the prediction process as CF’s concept, and Converting the neighbor rating range into the target user preference aspect. Therefore, this work proposed applying the neighbors’ concept of CF into a NN and imitating the CF’s rating prediction equation. Therefore, the proposed method consists of two modules toward the neighbors and the prediction module. The two toward the neighbor modules are the *similarity module* and *rating conversion module*, which can solve the similarity and rating conversion issues. The rating conversion module aims to obtain the neighbors’ ratings in the target user’s aspect, which is one key of the CF’s technique that needs to be concerned. To obtain the neighbor’s ratings in the target user aspect, neighbors’ vectors are used to project with the target user aspect matrix. Afterward, these neighbors’ vectors and the item’s vector are combined using the MF technique to obtain the neighbors’ ratings in the target user aspect. The similarity module aims to capture the neighbors’ attentions to obtain the similarity levels between the neighbors and the target user. These attentions can be obtained using a dot product between a neighbor and the target user pair. Both the rating conversion and similarity module outcomes are integrated into the prediction module. The prediction module uses a weighted average formula to imitate the CF’s prediction equation, where the similarities are used as a weight. There are four evaluation objectives in the experiment: 1) to test whether the proposed method performs better than the current NCFs with friends and the latent factor models, 2) to evaluate the effectiveness of our rating conversion in the proposed method, 3) to evaluate the performance of number of neighbors on real-world dataset, and 4) to compare the efficiency of the different data distributions and dataset size in the synthetic dataset. To evaluate the proposed method, two types of evaluation metrics are used: ranking-based evaluation, including normalized discounted cumulative gain (nDCG) and hit ratio (HR); and prediction accuracy metrics, including precision, recall, and root mean square error (RMSE). In the experiment, the real-world and synthetic datasets are used to evaluate the proposed method. To evaluate the effect of the number of neighbors, we divide the real-world dataset into two types: using *N* neighbors and all neighbors. We also need to know the efficiency of the rating conversion module. Therefore, the proposed method and the proposed method without rating conversion are compared. The synthetic datasets are used to evaluate the effect of data distribution and the dataset size using an ideal rating matrix. Our proposed method is compared with the current NCFs with friends methods and the two latent factor models using real-world and synthetic datasets. The results demonstrate that the proposed method significantly outperforms when compared with all baselines. Therefore, the considering similarity and the rating conversion of neighbors on neural collaborative filtering has contributed as follows.

- The rating conversion module transforms the neighbor’s aspect to the target user’s perspective, which has not been considered till now. It can solve the rating range issue between the neighbor’s rating range and the target user rating range by projecting the neighbor’s characteristics with the target user’s perspective view.
- The similarity module can detect the similarities level between the neighbor and the target user directly through the neighbors’ attentions. The neighbors’ attentions are captured using a dot product between the neighbor’s representation vector and the target user profile. Afterward, the neighbors’ attentions are normalized using a softmax function.
- There are three main ideas to complete in applying CF with NN: the similarity between the neighbor and the target user, the neighbor’s rating, and the rating conversion. Usually, the existing models consider only the similarity values or only neighbor’s ratings. In comparison, our proposed method concerns all of three main ideas.

## 2 Materials and methodology

### 2.1 Materials

Applying the MF [8] is one of the most commonly used techniques in the current RS approaches. For example, PLF [13] applied the MF technique to extract users’ latent factors and items’ latent factors from the rating matrix. After obtaining latent factors of users and items, the Pearson correlation is used to construct similarity values of users and items. The user’s and item’s neighbors are then selected based on their similarity values. The neighbors and similarity values are included in the latent factors of users and items. The MF approach is then used to integrate the latent factors of users and items to predict item ratings. In summary, PLF involved the MF technique directly and improved the user’s latent factor and item’s latent factor using similarity value and neighbor.

Previously, some research tried to utilize a neural network into the model, such as AK-UCF [19]. AK-UCF presented a combination of auto-encoder and *k*-means clustering to create a recommendation list. The auto-encoder network integrates user information, such as gender, address, age, and occupation, with a user preference to perform a user profile. Moreover, this network also decreases the dimension of user profiles vector to be suitable for *k*-means clustering. The *k*-means clustering part employed to allocate users based on their profiles. After that, the similarity values between users who belong to the same cluster are calculated. These similarity values are combined with the rating of users who belong to the same cluster with the target user directly to create a recommendation list. Although AK-UCF attempted to incorporate a NN into the CF concept, a neighbor part is not combined into a NN. This means AK-UCF used a NN to create user and item embedding only. Therefore, this research applies a clustering approach to create similarity values between users separately. Thus, their work is a semi-Neural CF, not a real Neural CF model.

In this subsection, four major topics are discussed: NCF without neighbors’ concept, NCF with the neighbors’ concept, rating conversion, and region embedding.

#### 2.1.1 Neural CF without neighbors’ concept

A few years ago, utilizing MF through a NN was widely used in the RS model, such as in the representation step, which initializes user and item vectors in the prediction step to compute the rating prediction and to decrease the dimension of the matrix in the system.

To represent the user and item as a vector, many current studies use a MF NN. First, the user and item vectors are initialized. Then, the MF concept is used to compute predicted rating (${\hat{r}}_{p_{u},q_{i}}$) for learning and adjusting the user and item vectors:

$$\begin{array}{c} {\hat{r}}_{p_{u},q_{i}}=p_{u}⊙q_{i}, \end{array}$$ (2)

where ⊙ denotes the element-wise product between two vectors. *p*_*u*_ and *q*_*i*_ are the user profile and the item characteristic vector, respectively. The target user’s actual rating values on the target item are labeled for the backpropagation method in the learning process. The number of layers and the hidden layer dimensions in this NN must be adjusted appropriately with the data and input dimensions. After learning the target users’ rating, each initialized user vector and item vector are improved according to each user profile and item characteristics. NCF [20] and temporal CNN for reviews based on the RS (TCR) [22] are examples of the NCF without neighbors concept.

NCF proposed three models: the GMF model, the MLP model, and the fusion of GMF and MLP called the neural MF (NeuMF) model. This work uses the one-hot vector to compute the model’s input, which is user and item representations. The GMF model is the MF method, which predicts rating value using an element-wise product between the user and item vector. The outcome of the GMF model is the predicted rating (*φ*^*GMF*^) of the target user on the target item. The MLP model also uses user and item vectors. The user and item vectors are combined using concatenation to learn the interaction between users and items. *φ*^*MLP*^ is the interaction vector that is the result from MLP network. The NeuMF model is the model that combines the GMF model and the MLP model. The GMF and MLP compute the rating value and the interaction vectors, respectively. The outcome of the GMF and the interaction vectors from MLP are merged into one vector using a concatenation operation in each user and item pair. Then, these concatenated vectors are learned in the last NN layer to create the predicted rating value *r*_*ui*_ and labeled by the target user’s actual rating on the target item:

$$\begin{array}{c} {\hat{r}}_{ui}=\sigma (h^{T}[\begin{array}{c} \varphi^{GMF}\\ \varphi^{MLP} \end{array}]), \end{array}$$ (3)

where *h* and *σ* denote weight and activation function of the NeuMF network output layer. *r*_*ui*_ is the predicted rating of the target user *u* on the target item *i*. Therefore, their work propose a framework that learns the rating and user’s interaction of NCF using only MF and the user’s interaction. However, there is no concept of neighbors in CF, which uses the neighbor’s preference for recommending items to the target user.

ONCF [21] improves NCF by learning the pairwise correlations between users and items by CNN instead of MLP. These correlations are the ratings of the users, given by the outer product between the users’ embeddings and the items’ embeddings. Their work aims to increase the efficiency of learning correlations between users and items. However, learning the rating matrix via CNN still lacks in leveraging the CF’s concept.

Because the user preference changes over time, the TCR model applies a time model in a CNN model. The TCR model consists of two networks: the user network and the item network, which execute the same process independently. Their work uses reviews from the users to extract the latent user profiles and item characteristics using the embedding method [27, 28]. Afterward, the user profiles and item characteristics are used as the input of the next layer, which is a convolutional layer and max-pooling layer. In the prediction step, both the user profile vector and item characteristic vector are used to compute the predicted rating vector using the MF technique (1). However, the comments or reviews can represent a user preference but cannot directly represent a user characteristic. Some of the comments that cannot represent a user characteristic include “fast delivery,” “Good quality bag,” and “Shoes are normally small on me so I went a size up and they fit great.” Moreover, this general framework does not consider the target user’s neighbors, which is a key CF technique.

#### 2.1.2 Neural CF with neighbors’ concept

Some researchers have applied the concept of neighbors into an NN such as NNCF [23], BPAM [24], the CMN [25] and SAMN [26].

The NNCF aims to integrate the neighbors’ information into the NCF model by concatenating the rating with the user’s neighbor information vector and the item’s neighbor information vector. These neighbor information vectors are performed by max-pooling each feature of all neighbor’s vectors. However, just adding the information using the concatenation operation into the ratings without using the similarity between the neighbor and the target user is not enough for the concept of CF technique.

BPAM proposed using the local weight and global attention weight to learn the rating of the target user. The local weight is the impact of the target user on each neighbor. The global attention weight is used as the impact on the target user on a set of neighbors. Both the local weight and global attention weight are combined and trained to predict the rating score. Although their work uses the similarity between the neighbors and the target user, it does not use the neighbors’ ratings into the prediction process as CF’s concept.

CMN tries to capture the user’s similarity and enhance the neighbor component in the NN attention mechanism. There are three steps: embedding, neighborhood attention, and rating prediction. First, the user history records are represented as a vector and form a user preference. Second, the new target user representation is obtained, which combines the neighbor component and the user embedding. The attention weights that infer the neighbor’s importance are fused into a NN layer to obtain the final neighbor’s representation. Finally, the target user’s rating score is produced via the MF concept using the target user’s and the target item’s representation. Then, the final neighbor’s representation and the target user’s rating scores are learned using a skip-connections NN. The CF’s prediction equation uses weighted average using the similarity among users and the neighbor’s rating scores where the similarity is used as a weight. However, CMN uses the neighbors’ representations to learn with the target user rating scores instead of the similarity level as in the CF concept.

SAMN proposes neighbor prioritizing, which considers the level of user preference. Because each neighbor of the target user has different expertise on different target items, Chen et al. [26] tried to create a user profile more accurately by fusing neighbors’ influence into the target user embedding. First, the user–item ratings are represented as user–item embeddings. The target user embedding and their neighbors embedding are combined through a joint embedding process to obtain an attention vector between the neighbor and the target user. Afterward, a fully connected layer is used to learn the attention score and normalize using a softmax function. To compute the friends’ vectors, the neighbor’s vectors are multiplied with their attention vectors dependent on the target user. Then, the friend-level attentions are computed and used to modify the target user embedding. Finally, the prediction score is computed using a MF between the modified target user embedding and the target item embedding.

Both current NCFs with friends, CMN and SAMN, give weight to the target user’s neighbor by combining them with the neighbor embedding. However, neighbor embedding is the representation of a user profile in terms of a numeric vector. Combining the weight with the neighbor embedding makes the user representation change, meaning it will cause the users’ representation to deviate from the real user profile and influence incorrect predictions. The neighbor’s weight should apply to the user’s rating for computing the target user’s predicted rating on the target item as in the CF rating prediction Eq (4):

$$\begin{array}{c} r\left(i,j\right)=\frac{\sum_{n\in N}sim\left(i,n\right)\cdot r_{n,j}}{\sum_{n\in N}|sim(i,n)|}, \end{array}$$ (4)

where *r*(*i*, *j*) denotes the predicted rating of item *j* for the target user *i*, *sim*(*i*, *n*) is similarity between the target user *i* and user *n*, *N* is the set of neighbors, and *r*_*n*,*j*_ is the rating of user *n* given to item *j*.

The Table 1 shows the comparison of the neural based CF’s related works. “✓” represent the presence of the neighbor concept in each column and “X” represent the absence of the neighbor concept. “-” means that there is no comparison. In summary, PLF is an MF-CF model rather than Neural CF because their work applied only traditional CF with MF, which did not involve any NN in the model. AK-UCF utilized a NN to construct user’s and item’s embedding. However, a neighbor part is created using a clustering approach separately. Therefore, AK-UCF is a semi-Neural CF model. The NCF, ONCF, and NNCF applied the neural network with the CF technique but did not use the neighbors of the target user to predict the rating. BPAM, CMN, and SAMN proposed using the neighbors and performing the similarity between the neighbors and the target user differently. However, they did not create and apply the neighbors’ ratings in the prediction process as the CF’s prediction equation. Moreover, no research converts the neighbor’s preference range into the target user preference aspect. The proposed method converts the neighbor’s preference range into the target user aspect and apply the neighbor’s concept using the similarity value and the neighbor’s rating.

**Table 1:** Comparison of the related works with respect to the neighbor concept.

	Non-Neural CF	Semi-Neural CF	Neural CF			
			Using neighbors	Calculating similarity	Applying the neighbors’ ratings in the prediction process	Converting preference range
**PLF**	✓	-	-	-	-	-
**AK-UCF**	-	✓	-	-	-	-
**NCF**	-	-	X	-	-	-
**ONCF**	-	-	X	-	-	-
**NNCF**	-	-	✓	X	X	X
**BPAM**	-	-	✓	✓	X	X
**CMN**	-	-	✓	✓	X	X
**SAMN**	-	-	✓	✓	X	X
**Proposed Method**	-	-	✓	✓	✓	✓

#### 2.1.3 Rating conversion

In the CF concept, the neighbors’ rating is used to predict the target user’s rating directly. However, the neighbors’ rating and the target user rating are in different ranges. Considering the neighbors and the target user preferences must use the same range. Thus, the method that converts the neighbor’s rating pattern into the target user’s rating pattern is called rating conversion. For example, suppose that the rating range is 1–5. User A gives ratings in the 1–3 range for “dislike” to “like,” while user B gives ratings in the 3–5 range instead. This means that “like” of A (rating 3 of A) equals “dislike” of B (rating 3 of B). Thus, using one user’s rating to predict another user’s rating directly is not good.

Although some researchers tried to solve the rating conversion issue using the normalization technique that maps every user’s rating into a range from 0 to 1, it is still not effective enough. If two target users have the same group of neighbors, both target users will receive the same predicted rating or the same recommendation. For example, target users A and B usually have normalization rates of 0.4 and 0.7, respectively, and the predicted ratings of both the target users on the target item are 0.8. Therefore, this target item should be recommended to user A rather than user B because the predicted rating of the target item is in the range that user A likes, while it is in the average range of user B. The W’s transpose function [29] is proposed to solve the rating conversion issue; it transposes the user’s rating into the target user’s aspect using a relation between both users’ rating patterns. There are four key terms in the W’s transpose function, which used to convert the user’s rating: original value, adjustment, confidence, and distribution. The original value is the rating that will be transposed. The adjustment part is the average difference of co-rated items between the user and the target user. The confidence term and distribution term are added to obtain more accurate results.

#### 2.1.4 Region embedding

**Fig 1:**
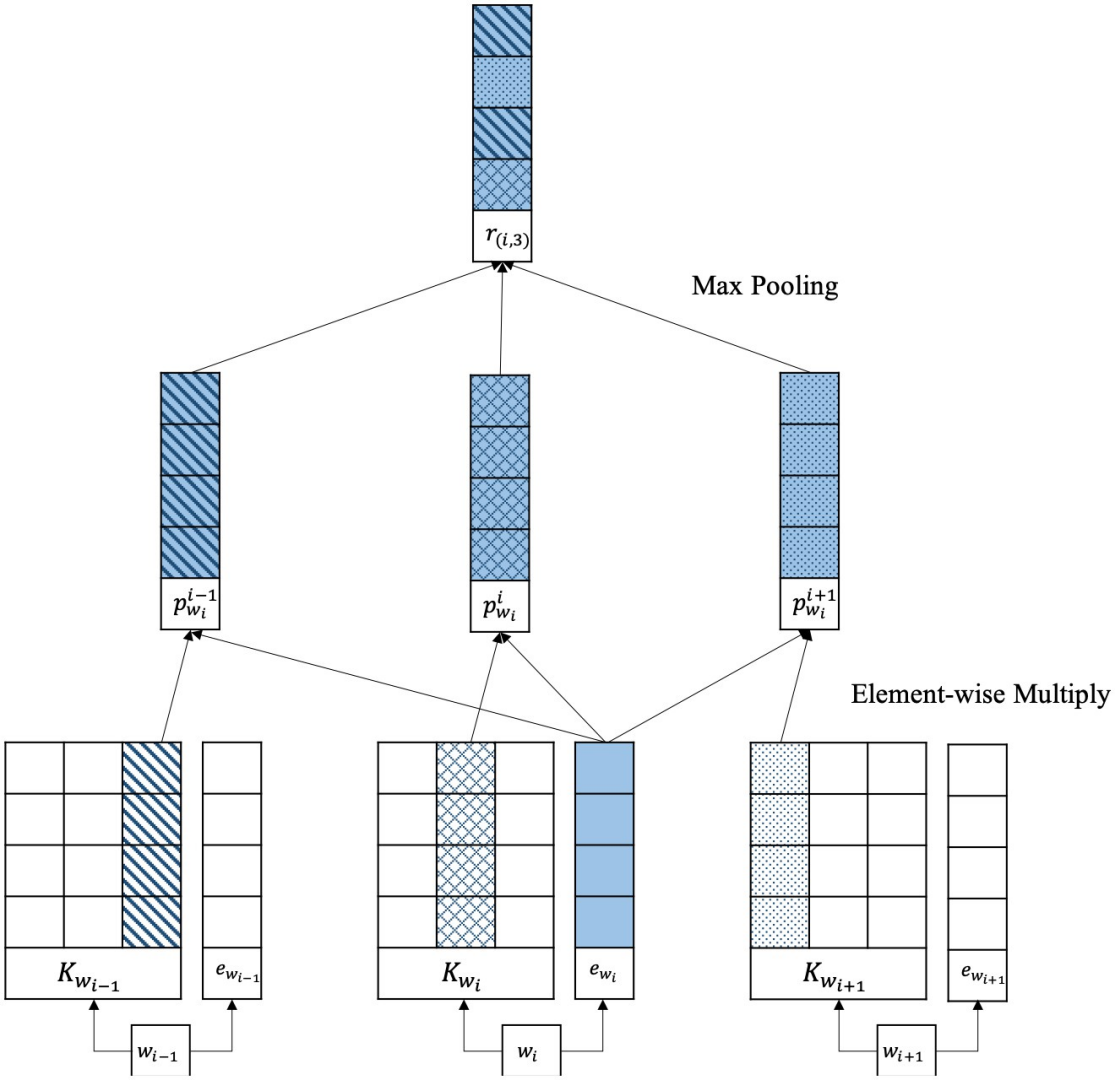
Context-word region embedding method.

Recently, a new embedding technique called the region embedding method that uses the words in the same region to obtain the word representation was proposed [30]. It is the assumption that one embedded word should not be in the same representation in a different region of the document. It is a representation of the continuous subsequence of the words in the document called the text region. Moreover, this method can solve the sparsity problem of the *n*-grams method. The text region, for example, given a sentence “The story is sweet and simple and easy to read,” a region of length five means “story is sweet and simple.” The region embedding method consists of three steps, as shown in Fig 1, to obtain the region embedding vector.

First, embed input words in the selected region to produce the target word embedding vector and local context unit (LCU), a word’s weight matrix in this region. Both the target word embedding vector and LCU are produced using the embedding method. Second, project the LCU into the target word embedding vector using the element-wise multiplication operation:

$$\begin{array}{c} p_{w_{i+t}}^{i}=K_{w_{i,t}}⊙e_{w_{i,t}}, \end{array}$$ (5)

where $p_{w_{i+t}}^{i}$ is the projected word embedding of *w*_*i*+*t*_ in *i*_*th*_ word, $K_{w_{i,t}}$ is LCU, and $e_{w_{i,t}}$ is the embedding of word *w*_*i*+*t*_. Afterward, the projected word embeddings, which are from the previous step, are combined using max pooling to perform the word representation.

### 2.2 Methodology

#### 2.2.1 Overview of the proposed method

In region embedding, the word that is in the same region as the target word is called the LCU. For example, if the target word “Apple” is in the two regions with the LCU1 and LCU2 for region1 and region 2, respectively, the word “Apple” is represented as “Apple 1” and “Apple 2,” respectively. Because of the difference between the relation of the target word and each LCU, the representation of the target word is different in each region.

**Fig 2:**
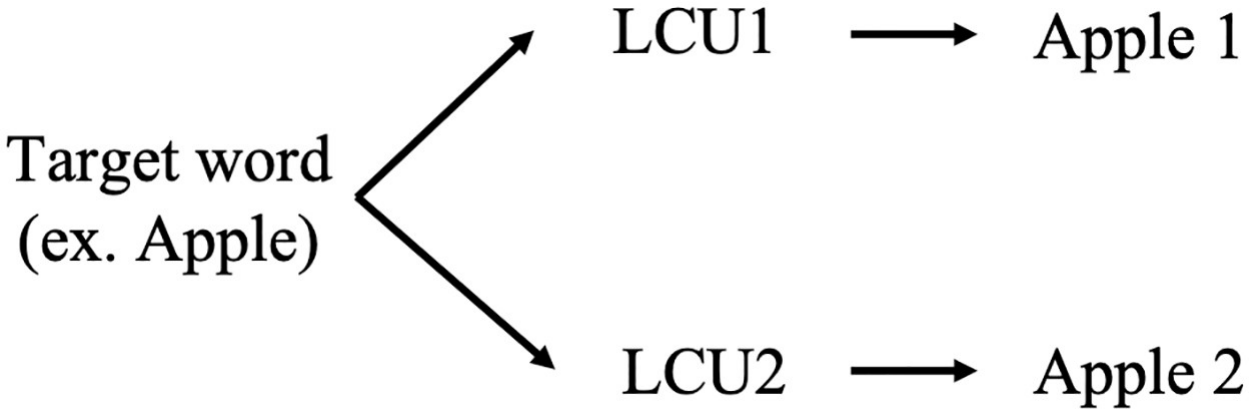
Context unit example.

When applying the region embedding assumption with the CF concept, the target user is viewed as a target word. The neighbors of the target user are viewed as the LCU of the target word. Therefore, the target user will give the different predicted ratings for the two different groups of neighbors, similar to the “Apple 1” and “Apple 2” in the region embedding example (Fig 2).

In CF, the concept of neighbors is integrated into the model in the prediction step. The neighbors’ rating scores and the similarity levels between the neighbors and the target user are used to predict the target user’s rating score as in (4). In the past, no NCF research used neighbors’ ratings in the prediction process as in CF. When applying a NN with the CF, two issues need to be considered: the similarities between the neighbors and the target user and the rating conversion. The region embedding assumption is used to solve the rating conversion issue, converting the neighbors’ ratings range into the target user rating range. The similarity issue is solved by capturing neighbors’ attentions to compute the similarity level between the neighbors and the target user.

**Fig 3:**
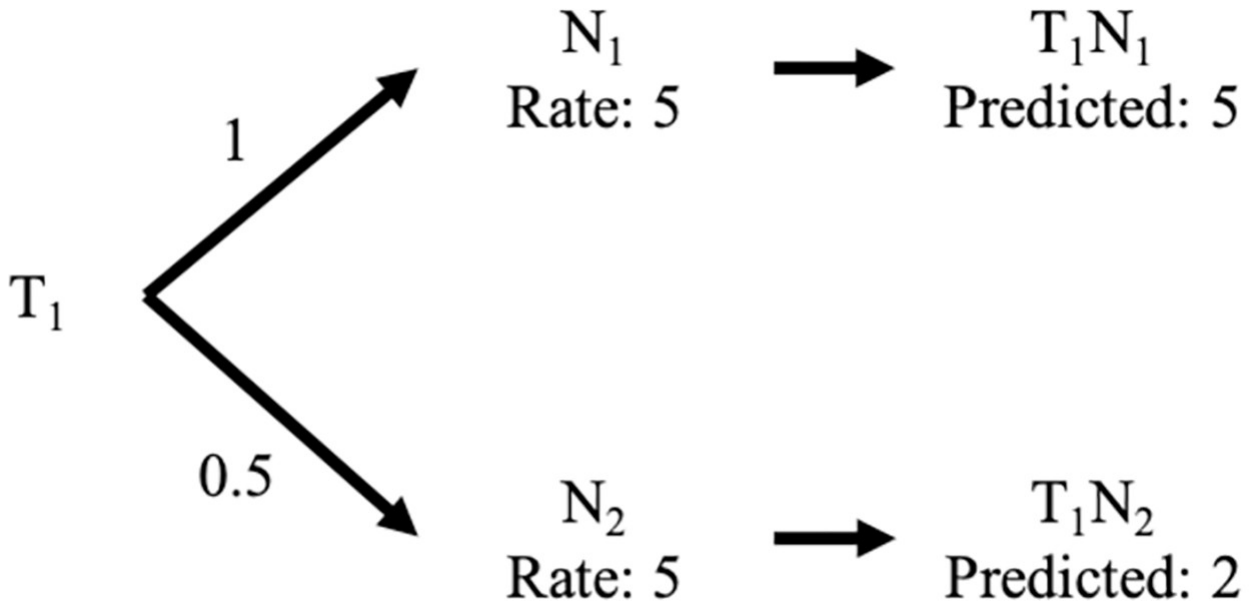
Example of similarity among users.

In the case of similarity issue: the neighbors’ concept is the CF technique’s key idea, which uses the neighbors’ preferences to compute the similarity levels between the neighbors and the target user and the rating prediction. To compute the similarity, many techniques are used, such as cosine similarity and Pearson’s correlation, to determine the neighbors’ preferences. The similarity levels between the neighbors and the target user are used to weigh the neighbors’ ratings to predict the target users’ ratings. For example, a rating range of 1–5 for “dislike” to “like” and a similarity range of 0–1 for “not similar” to “similar.” The target user has two neighbors, *N*_1_ and *N*_2_, in Fig 3. The similarities between the target user and neighbors *N*_1_ and *N*_2_ are 1 and 0.5, respectively. If both neighbors gave a score of 5 to the target item, but the similarity value is different, the rating from *N*_1_ and *N*_2_ is transferred to the target user depending on the similarity level of *N*_1_ and *N*_2_. Therefore, the target user’s rating predictions via *N*_1_ and *N*_2_ are 5 and 2.5, respectively.

**Fig 4:**
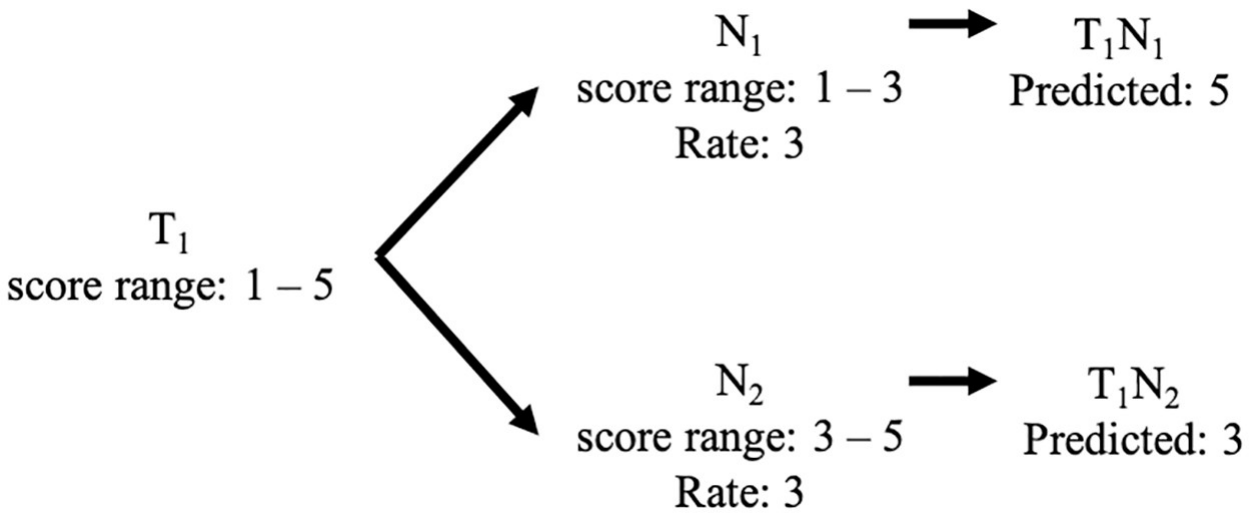
Example of rating conversion issue.

In the rating conversion issue, because of different users having different rating ranges, the neighbors’ ratings are mapped from the neighbors’ rating range to the target user’s rating range. For example, the target user gives ratings in the range of 1–5 for “dislike” to “like,” while *N*_1_ gives ratings in the range from 1 to 3 and *N*_2_ gives ratings in the range from 3 to 5 instead. Suppose both neighbors gave scores of 3 to the target item, as shown in Fig 4. If the rating conversion is not considered, scores of 3 from the neighbors will map directly to the target user, which means neutral for the target user. However, *N*_1_ wants to tell the target user that the target user should like the target item, which is a score of 5 for the target user, and the *N*_2_ wants to tell that the target user should not like this item, which is a score of 1 for the target user. Therefore, the rating conversion should be considered in the model.

**Fig 5:**
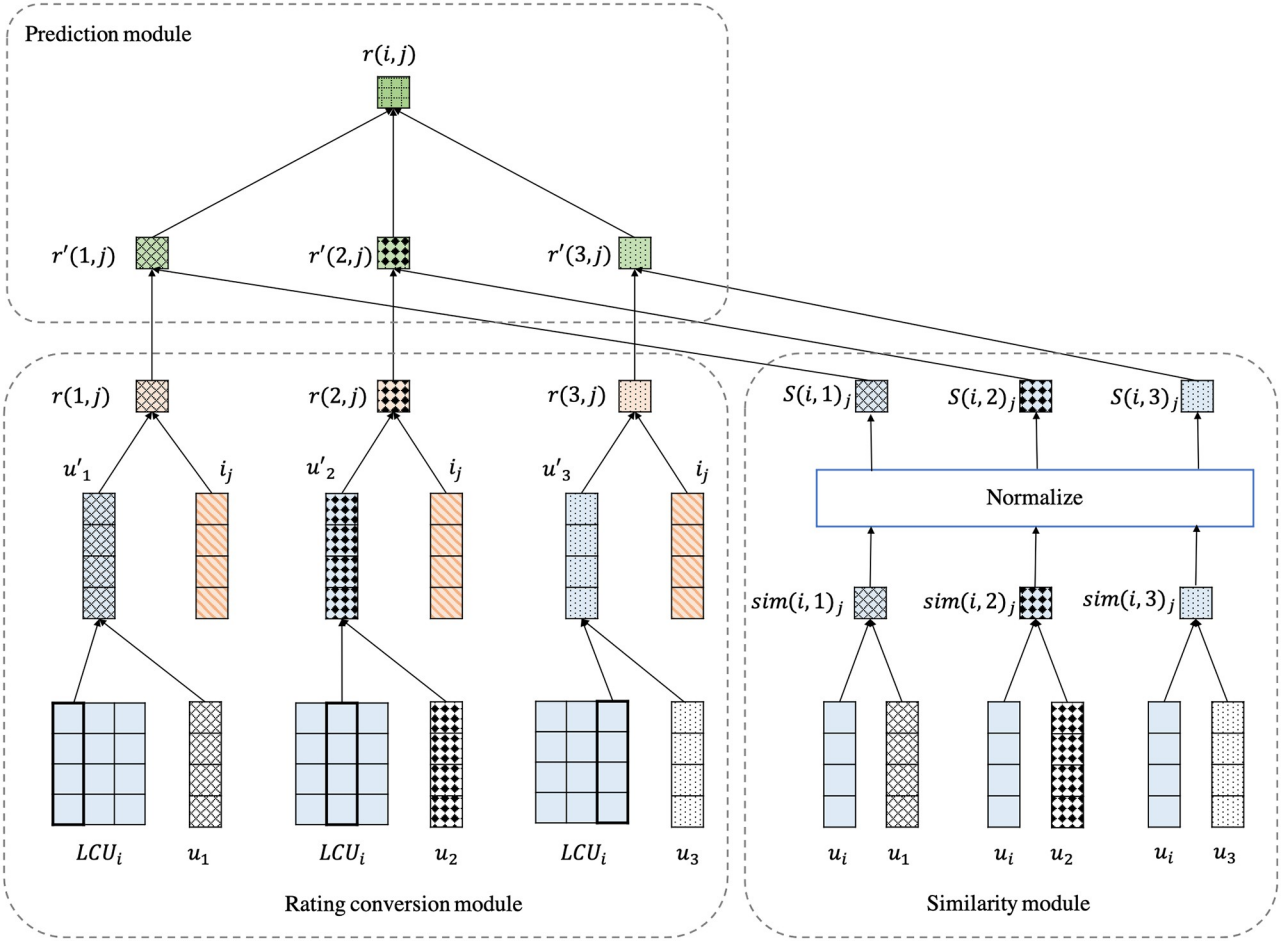
The proposed model architecture.

To solve these two issues, we propose three modules: the rating conversion module, the similarity module, and the prediction module. The rating conversion module projects the neighbor’s characteristics on each target user’s perspective view. In other words, the neighbors will be changed into the term of target user aspect, which is similar to the LCU concept of the region embedding. The similarity module captures the neighbors’ attentions to compute the similarity levels between the neighbors and the target user. The prediction module uses the rating conversion and the similarity module outcome to imitate the CF’s prediction equation. The architecture of the proposed model is shown in Fig 5.

#### 2.2.2 Steps of the proposed method

The proposed method consist of five steps: selecting neighbors, user–item embedding, converting neighbors into the target user aspect, crating similarity, and rating prediction.

*2.2.2.1 Selecting neighbors*. In the CF technique, using the neighbors’ concept to predict the target user’s rating on the target item is crucial. To select *N* neighbors of each target user, the group of rater who rated the target item are chosen. If the number of rater is less than or equal to *N*, all rater users are selected as neighbors. If the number of rater users is greater than *N*, the *N* rater are selected randomly to be the neighbors of the target user. Therefore, the outcome of this step is a list of *N* neighbors.

*2.2.2.2 User–item embedding*. After preparing the neighbors’ list, the inputs of the proposed method are the target user, the target item, and the list of neighbors. In each history record, the user and item pair are initialized in the same embedding size to represent the target user and the target item. The neighbors’ list of the target user is initialized as a matrix called the neighbors’ matrix, and each row is the neighbor’s embedding. If the number of the rater is less than *N*, the weights of all rater users’ in the NN model are initialized. The remaining neighbors’ weights are assigned zero. For example, the number of neighbors is set to 10. Therefore, 10 neighbors’ embeddings are initialized. Suppose the number of rater users is six, which is less than 10, then model’s weights of six neighbors are initialized. The remaining four weights are assigned as zero, meaning these four neighbors’ embedding will not be learned. The target user vectors, the target item vectors, and neighbors’ matrix will be adjusted to represent the target users, items, and neighbor characteristics using the target user ratings as a label of the model.

*2.2.2.3 Converting neighbors into the target user aspect*. To solve the rating conversion issues, the LCU concept in the region embedding [30] is applied. In region embedding, the LCU is the influence of each word in the region on the target word, which is used to obtain the target word representation in that region. In the same way, we apply the LCU with the neighbor concept by learning LCU via the target user ratings and use as the target user aspects on the neighbors to generate the neighbor representation in the target user’s perspective view. Therefore, we propose the rating conversion module in Fig 5, in which the neighbor’s preferences are converted into the target user aspect. First, the LCU of target user *i* (*LCU*_*i*_) is initialized in embedding the size-by-number of the neighbors’ matrix. In Fig 5, suppose there are three neighbors and four elements of embedding size. Therefore, the dimension of the target user’s LCU is a four-by-three matrix. Afterward, the target user’s LCU on the neighbor *n* (*LCU*_(*i*, *n*)_) is combined with the neighbors’ embeddings (*u*_*n*_) using the element-wise multiplication to obtain the projected neighbors’ representations in the target user perspective ($u_{n}^{\prime }$).

$$\begin{array}{c} u_{n}^{\prime }=LCU_{\left(i,n\right)}⊙u_{n} \end{array}$$ (6)

To compute the neighbors’ predicted rating scores in the target user aspect (*r*_(*n*,*j*)_), these projected neighbors’ representations and the target item’s embedding (*i*_*j*_) are integrated together using MF technique (7).

$$\begin{array}{c} r_{\left(n,j\right)}=u_{n}^{\prime }\cdot i_{j} \end{array}$$ (7)

To adjust the target user’s LCU, neighbors’ embeddings, and other parameters in the model, the target user ratings are labeled and learned using the backpropagation algorithm.

*2.2.2.4 Creating similarity*. Usually, the similarity levels between the neighbors and the target user are computed from cosine similarity or Pearson’s correlation equation on a pair of the target user and each neighbor. In this work, the similarity levels between the neighbor and target user are attentions between two users. To compute attention among users, a pair of the target user vector (*u*_*i*_) and the neighbor vector ($u_{n}^{\prime }$) are combined using a dot product.

$$\begin{array}{c} sim{\left(i,n\right)}_{j}=u_{i}\cdot u_{n}^{\prime } \end{array}$$ (8)

Afterward, the softmax function (9) is used to adjust the similarity value into the range [0, 1]. This step’s architecture is shown in the similarity module in Fig 5.

$$\begin{array}{c} S{\left(i,n\right)}_{j}=\frac{exp(sim{\left(i,n\right)}_{j})}{\sum_{n\in N}exp\left(sim{\left(i,n\right)}_{j}\right)} \end{array}$$ (9)

*2.2.2.5 Rating prediction*. In the CF approach, the concept of neighbors is integrated into the system in the prediction step, in which the neighbor’s ratings are used to predict the target user’s rating. The same as the CF approach, the rating prediction Eq (10) is imitated into the proposed method in this step using the neighbor’s predicted rating scores and the similarities between the target user and their neighbors. As the rating prediction formula, the neighbors’ rating (*r*(*n*, *j*)) needs to be weighed because each neighbor has a different similarity level toward the target user. Therefore, the target user’s predicted rating scores are computed using a weighted average on the neighbors’ ratings where the similarity levels are used as a weight. The result of the proposed method is the predicted rating of the target user *i* on the target item *j* (*r*(*i*, *j*)). The prediction module in Fig 5 is the architecture of this step.

$$\begin{array}{c} \begin{array}{cc} r^{\prime }\left(n,j\right) & =r\left(n,j\right)\cdot S{\left(i,n\right)}_{j},\\ r\left(i,j\right) & =\frac{\sum_{n\in N}r^{\prime }\left(n,j\right)}{\sum_{n\in N}S{\left(i,n\right)}_{j}} \end{array} \end{array}$$ (10)

Finally, we calculate the time complexity for one epoch to train the NN. Due to the proposed method performed by three layers, the time complexity of feedforward and back-propagation is *O*(*e*_*n*_ *e*_*cn*_ *t*), where *e*_*n*_, *e*_*cn*_, and *t* are the neighbor embedding size, the neighbor in the target user aspect embedding size and the number of neighbors, respectively. In this work, the neighbor embedding and the neighbor in the target user aspect embedding are the same sizes. Due to training the model, the number of epochs in each dataset is different. The time complexity of our proposed method also depends on the number of epochs. Therefore, the time complexity of the proposed method depends on the neighbor embedding size, the number of neighbors, and the number of epochs.

## 3 Evaluation

In the real world, users’ interactions are collected in a variety of formats such as ratings, reviews, images. The format that is widely used for evaluating the RS model is the ratings of users on items. However, using real-world datasets unable to control the data distribution Therefore, the proposed method uses both real-world and synthetic datasets in the evaluation. Our proposed method is compared with the existing methods current NCF with friends, which are CMN [25] and SAMN [26]. Furthermore, we want to know our model can beat the latent factor models or not. The latent factor models that are used in the evaluation are the Singular Value Decomposition (SVD) factorizes and Non-negative matrix factorization (NMF). The neighbor is important in CF research. Therefore, we would like to know the performance of using a difference number of neighbors, *N* neighbors and all neighbors. Moreover, the effectiveness of our rating conversion of the proposed method is evaluated. The proposed method is compared with the proposed method without the rating conversion, in which the target user’s LCU is removed from the rating conversion module. In other words, the neighbors’ representations without the rating conversion are used to generate the neighbors’ ratings directly via applying the MF concept.

For the synthetic datasets, we also evaluate the proposed method with the SVD, NMF, CMN, and SAMN. In order to simulate the datasets, we generate the full rating matrix and partial rating matrix to compare based on normal rating distribution. The full rating matrixes are created in different rating distributions and sizes. The partial rating matrix is a rating matrix that is similar to the real world but in the normal rating distribution. Usually, GMF and NCF are two famous baselines. However, both CMN and SAMN have been demonstrated to outperform GMF and NCF. Thus, GMF and NCF will not be compared in this work. To evaluate the proposed method, we select the ranking-based evaluation and prediction accuracy to compare the accuracy and the ranking performance.

### 3.1 Real-world datasets

For the real-world dataset, we select the different categories of datasets to show that the proposed method can be applied to various categories, movie, online product, and restaurant.

#### 3.1.1 Data preparation

To evaluate the performance of the proposed method, three public datasets are used:

- *MovieLens 1M:* [31] This dataset is collected by grouplens. It contains one million movie ratings, is a stable benchmark dataset, and is widely used in research experiments. It contains the user ID, movie ID, users’ ratings, and timestamp. Moreover, grouplens provides at least 20 records per user.
- *Epinions:* [32] This dataset is a who-trust-whom online social network that contains product ratings and reviews. It contains the product name, category, and timestamp.
- *Yelp:* [33] This dataset contains users’ personal data and reviews, ratings, pictures, and details of business and services in four countries. It is released for the academic challenge and teaches students about databases.

The rating range of MovieLens 1M, Epinions, and Yelp dataset is [0.5,5]. Each dataset consists of four basic attributes: userID, itemID, rating, and timestamp. The number of records, users, and items of each dataset are shown in Table 2.

**Table 2:** The number of records, users, and item in each dataset.

Dataset	Records	Users	Items
MovieLens 1M	1,000,209	6,040	3,706
Epinions	664,824	49,290	139,738
Yelp	8,021,122	1,968,703	209,393

To ensure that each user has enough records represented as a vector, the users who have more than twenty records are selected in the preprocessing step. The number of records, users, and items after preprocessing are shown in Table 3. The MovieLens dataset provides at least twenty records per user. Thus, the number of records, users, and items in Table 3 are the same as in Table 2.

**Table 3:** The number of records, users, and items in each dataset after preprocessing.

Dataset	Records	Users	Items
MovieLens 1M	1,000,209	6,040	3,706
Epinions	564,709	8,217	106,242
Yelp	2,253,312	57,814	31,943

The statistics of each dataset have shown in Table 4, which consist of Mean, Median, Mode, and Standard Deviation (SD). From Table 4, the highest mean value goes to the Epinions dataset. That means the Epinions dataset contains the highest number of high rating scores compared with the Movielens and yelp datasets. The rating scores of all datasets have the same median value, which is four. Most of the rating scores in the Epinions and Yelp datasets are five, while the Movielens dataset is four. When comparing a standard deviation, the Yelp dataset contains the highest rating dispersion compared to the Movielens and Epinions datasets.

**Table 4:** Statistic of each dataset.

Dataset	Mean	Median	Mode	SD
**MovieLens 1M**	3.58	4.00	4.00	1.12
**Epinions**	3.97	4.00	5.00	1.20
**Yelp**	3.70	4.00	5.00	1.49

#### 3.1.2 Experimental settings

To evaluate the dataset, each dataset is randomly selected in the ratio 80:10:10 for training:validation:testing sets. The baseline parameters are used as in the corresponding papers. The proposed method’s batch size was tested in [32, 64, 128], and the learning rate was tested in [0.01, 0.05, 0.1]. To demonstrate the impact of the number of neighbors and embedding size, we fixed and evaluated the proposed model at nDCG@*k* for *k* ∈ {5, 10, 20, 30, 40, 50}. In this work, we tried to use an embedding size of 8, 16, 32, 64 and neighbor size of 10, 20, 30, 40, 50.

This work aims to apply a NN into the CF concept, which considers similarity levels between the neighbors and the target user and rating conversion. To optimize the model, we use the adaptive momentum (Adam) [34] estimation optimizer, which is widely used in current works and requires small memory. The Adam optimizer is a combination of root mean square propagation gradient descent (RMSprop) [35] and stochastic gradient descent (SGD) [36]. In the learning process, the Adam optimizer computed adaptive learning rates for each parameter and the exponentially decaying average of past gradients. We used the loss function in a multi-class classification task called categorical cross-entropy to minimize the prediction error and learn the model parameters:

$$\begin{array}{c} L=-\sum_{c=1}^{C}y_{\left(i,j\right)}\cdot \text{log}\;r_{\left(i,j\right)}, \end{array}$$ (11)

where *C* denotes the number of output classes, which is a number of rating classes. In this work, *C* is set at 10 or 5 depending on the range of rating in each dataset. *y*_(*i*,*j*)_ and *r*_(*i*,*j*)_ are the actual rating and the predicted rating of user *i* on target item *j*. After evaluating the impact of the number of neighbors and embedding size of the proposed method, we compare the proposed method with the other two methods using nDCG@*k*, HR@*k* for *k* ∈ {5, 10, 20, 30, 40, 50}, Precision, Recall, and RMSE.

#### 3.1.3 Evaluation metrics

To evaluate the proposed method, there are two types of evaluation metrics: ranking-based evaluation and prediction accuracy. Two ranking quality metrics are used in this work: nDCG and HR. In addition, we use precision, recall, and RMSE to indicate the prediction accuracy.

*3.1.3.1 Ranking-based evaluation*. The nDCG is one of the most popular metrics that evaluate the ranking quality using relevant score. The nDCG equation defined as follows:

$$\begin{array}{ll} DCG_{p} & =\sum_{i}^{p}\frac{2^{rel_{i}}-1}{log_{2}(i+1)},\\ IDCG & =\sum_{i=1}^{\left|REL_{p}\right|}\frac{2^{rel_{i}}-1}{log_{2}(i+1)},\\ nDCG_{p} & =\frac{DCG}{IDCG}. \end{array}$$ (12)

where *p* is a particular rank position, *rel*_*i*_ is the relevance of recommendation at position *i*, and |*REL*_*p*_| is the list of items ordered by relevance in the corpus up to position *p*.

The HR indicates the accuracy of recommendation top *k*. If the item in the top *k* recommendation list matches the item that the target user has rated in top *k*, it means “hit.” The number of hits divided by the number of all users in the test set is the HR value.

$$\begin{array}{c} HR=\frac{\text{number}\;\text{of}\;\text{hits}}{\text{total}\;\text{number}\;\text{of}\;\text{users}}. \end{array}$$ (13)

*3.1.3.2 Prediction accuracy*. To evaluate the prediction accuracy, we use three metrics: precision, recall, and RMSE. Precision is a measure of exactness. It is the proportion of the recommended items in the top *k* set that are relevant, which is the number of the recommended items that are relevant divided by the total number of recommended items. The precision is defined as follows.

$$\begin{array}{c} Precision=\frac{\text{relevant}\;\text{item}}{\text{recommended}\;\text{item}}. \end{array}$$ (14)

The recall is the proportion of the relevant item on a total number of relevant items, which is the number of the recommended items that are relevant divided by the total number of relevant items.

$$\begin{array}{c} Recall=\frac{\text{relevant}\;\text{item}}{\text{all}\;\text{relevant}\;\text{item}}. \end{array}$$ (15)

The predicted ratings that are higher than three scores are defined as relevant in this work.

Another way to evaluate the rating prediction accuracy is by measuring the error of the predicted rating. Therefore, RMSE is used. To calculate the RMSE, the predicted rating (*r*(*i*, *j*)) is compared with the actual rating ($\hat{r}\left(i,j\right)$). The summation of the error squared values is averaged over all predictions to obtain the overall RMSE:

$$\begin{array}{c} RMSE=\sqrt{\frac{1}{N}\cdot \sum_{i=1}^{n}{\left(r\left(i,j\right)-\hat{r}\left(i,j\right)\right)}^{2}}, \end{array}$$ (16)

where *N* is the total number of the prediction.

#### 3.1.4 Experimental results

The number of neighbors, who have rated the target item, is important in this work. Traditional CFs use the similarity between the neighbor and the target user to weigh the neighbor’s ratings. Therefore, the neighbors of the target user are essential in the CF research. In the proposed method, the neighbors are selected by two criteria: the users who have rated the target item (raters) and the similarity value of more than zero. In section 2.2.2.4, the attentions between the neighbor and the target user are normalized using the softmax function and the threshold to create the similarity value between the neighbor and the target user. The similarity between the neighbor and the target user in traditional CF’s technique is in the range [0, 1]. In this work, we select the raters who have similarity more than zero as the neighbors. The similarity between the neighbor and the target user is in range [0, 1] as in CF technique. This similarity value indicates how similar the neighbor is to the target user. Suppose the similarity value of the neighbor closes to one means this neighbor is most similar to the target user. In contrast, if the similarity value is close to zero, this neighbor is a low-quality neighbor. However, there is a large number of raters in each dataset. Using all raters as the neighbors can make the incorrect prediction because some neighbors are the noise of recommendation. In order to find the effectiveness of the number of neighbors, this work evaluates two types of neighbors usage: *N* neighbors and all neighbors. The *N* neighbors are the randomly selected *N* raters as neighbors. In contrast, all neighbors use all users who have rated the target item to be the neighbors.

**Fig 6:**
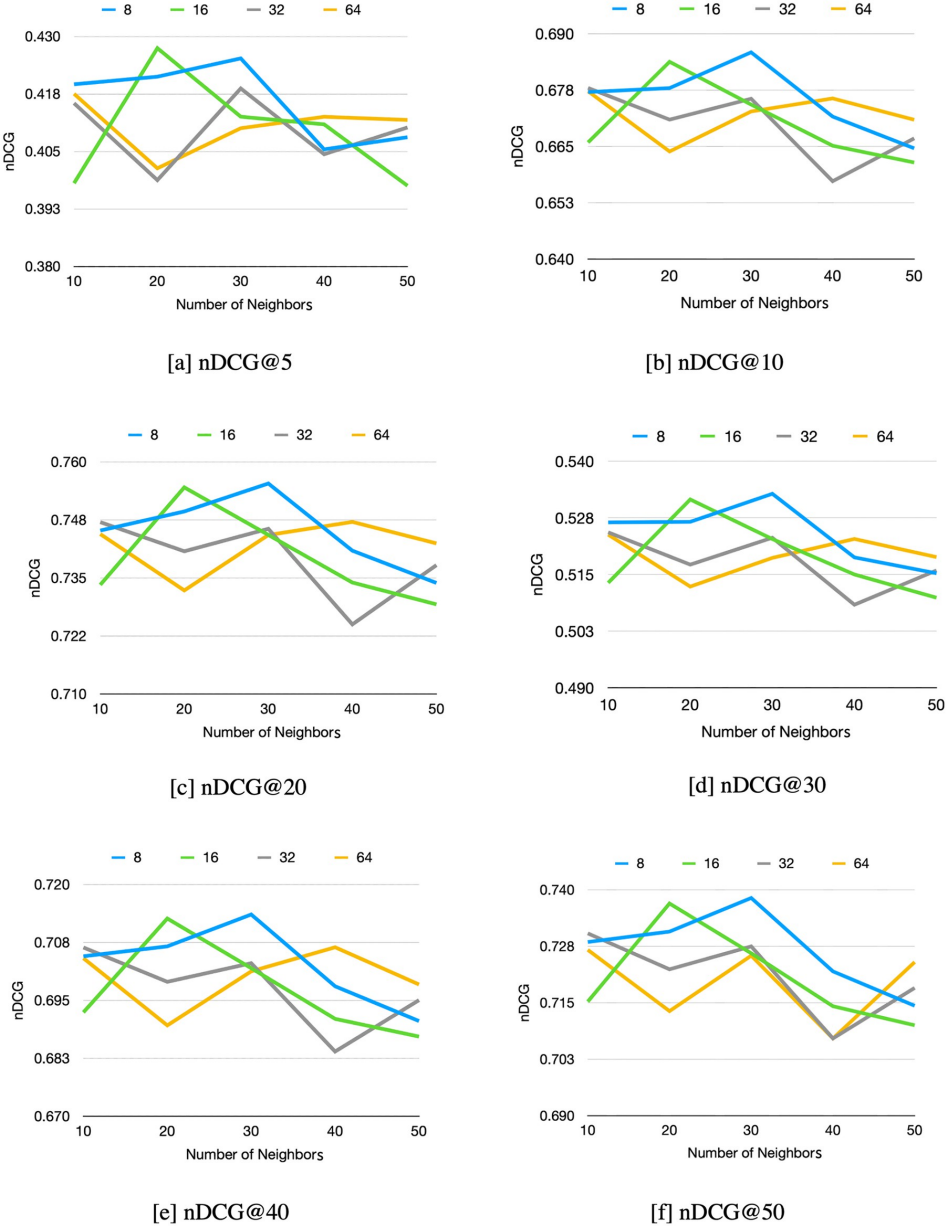
Experimental results of the Movielens 1M dataset for the proposed method varying the embedding size and neighbors. [a] nDCG@5. [b] nDCG@10. [c] nDCG@20. [d] nDCG@30. [e] nDCG@40. [f] nDCG@50.

*3.1.4.1 N neighbors*. The proposed method solves the two issues: the similarities between the neighbors and the target user and the rating conversion. The neighbors’ concept is considered and applied to the NN model, in which the neighbors are used to predict the preference of the target user. Because the similarity issues are directly implicated with the number of neighbors, it is an essential hyperparameter that needs to be tuned. The embedding size of the target user, neighbor, and item representations are other essential hyperparameters used to represent the user–item interaction profiles via ratings. The MovieLens 1M dataset’s experimental results are shown in Fig 6. Fig 6 shows the changes in the experimental results when using different embedding sizes and a different number of neighbors.

Fig 6[a]–6[f] shows that the embedding size of 8 obtained the highest nDCG results when the numbers of neighbors were 10 and 30 when comparing with the embedding sizes of 16, 32, and 64. Even though the embedding size of 16 provided the highest performance in Fig 6[a] when there were 20 neighbors, 10, 30, 40, and 50 neighbors gave lower nDCG results than the other three embedding sizes. In the same case as the embedding size of 16, the embedding size of 64 obtained the highest nDCG results when the number of neighbors was 50 in Fig 6[a]–6[e]. However, 10, 20, 30, and 40 neighbors at the embedding size of 64 obtained lower nDCG results than the other three embedding sizes. Although the embedding size of 32 obtains the same tends as the embedding size of 8, the embedding size of 32 provides a lower nDCG result than the embedding size of 8. The overall nDCG results obtained from embedding size 8 are greater than the other three embedding sizes, especially at a neighbor size of 30. In terms of the neighbor parameter, most of the nDCG results increase when the number of neighbors reaches 30. Afterward, the results decrease. The result shows that increasing the number of neighbors can improve the rating prediction accuracy. According to the experiment results, only the first 30 neighbors are high-quality neighbors, and the number of neighbors more than 30 neighbors are low-quality neighbors. If the number of neighbors is too high, the low-quality neighbors decrease the nDCG. Therefore, we selected an embedding size of 8 and neighbors 30 to compare with the CMN and SAMN on the MovieLens 1M dataset.

We tried to experiment with an embedding size of 8, 16, 32, and 64 and the number of neighbors 10, 20, 30, 40, and 50 on the Epinions and Yelp datasets. Moreover, the number of the neighbor parameter is varied in the same way as in the MovieLens 1M dataset on both datasets. The overall nDCG ranking results from the Epinions and Yelp datasets that obtain the best results are at embedding sizes of 64 and 32, respectively. Thus, the Epinions dataset at an embedding size of 64 and the Yelp dataset at the embedding size of 32 are used to compare with the proposed method.

**Fig 7:**
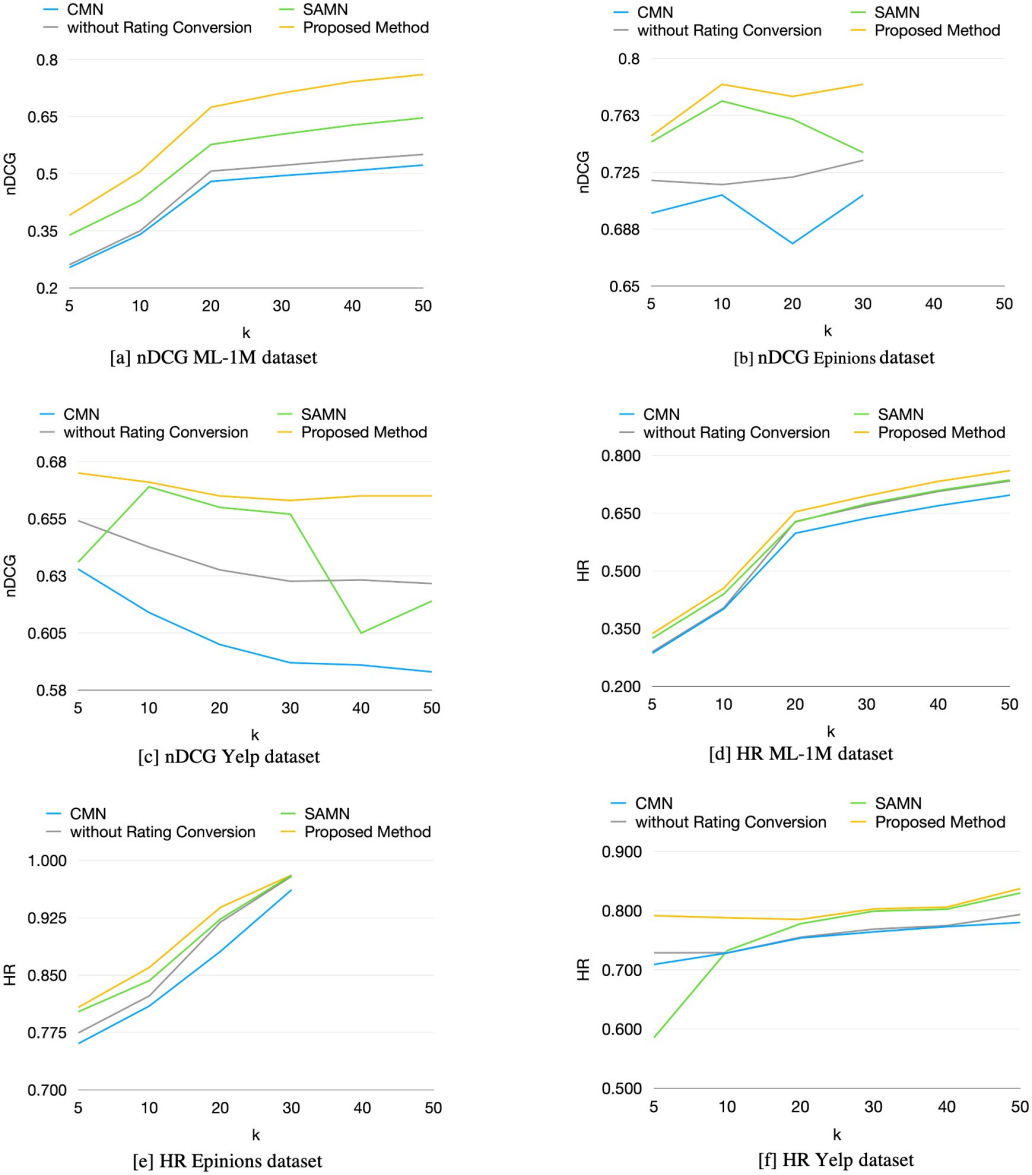
Experimental results graph comparison on the ML-1M, Epinions, and Yelp datasets using the nDCG and HR. [a] nDCG ML-1M dataset. [b] nDCG Epinions dataset. [c] nDCG Yelp dataset. [d] HR ML-1M dataset. [e] HR Epinions dataset. [f] HR Yelp dataset.

The comparison results of the proposed method’s results, latent factor models, and the current NCFs with friends using 30 neighbors at embedding sizes of 8, 64, and 32 on the MovieLens 1M, Epinions, and Yelp datasets using nDCG are shown in Table 5 and Fig 7[a]–7[c]. The HR experimental results are shown in Table 6 and Fig 7[d]–7[f]. The proposed method achieves significantly higher results than SVD, NMF, CMN and SAMN at all *k*. Although some of the proposed method results are slightly lower than the SAMN method, the proposed method takes much less processing time than the SAMN method, approximately three hours less on the MovieLens 1M dataset. The Epinions dataset is a small dataset when compared with the other two datasets. Therefore, this dataset does not have enough records to experiment on nDCG@40 and nDCG@50 after preprocessing.

**Table 5:** Comparison of the experimental results on the ML-1M, Epinions, and Yelp datasets using nDCG.

Datasets	Methods	nDCG@k					
		5	10	20	30	40	50
**ML-1M**	**SVD**	0.267	0.421	0.605	0.645	0.674	0.695
	**NMF**	0.244	0.390	0.580	0.619	0.649	0.667
	**CMN**	0.254	0.341	0.480	0.495	0.508	0.523
	**SAMN**	0.339	0.430	0.577	0.604	0.628	0.647
	**Without Rating Conversion**	0.261	0.350	0.507	0.522	0.537	0.551
	**Proposed Method**	**0.391**	**0.506**	**0.675**	**0.712**	**0.742**	**0.761**
**Epinions**	**SVD**	0.717	0.752	0.775	0.750	-	-
	**NMF**	0.700	0.747	0.767	0.761	-	-
	**CMN**	0.698	0.710	0.678	0.710	-	-
	**SAMN**	0.745	0.772	0.760	0.738	-	-
	**Without Rating Conversion**	0.720	0.717	0.722	0.733	-	-
	**Proposed Method**	**0.749**	**0.783**	**0.775**	**0.783**	-	-
**Yelp**	**SVD**	0.610	0.627	0.639	0.641	0.648	0.653
	**NMF**	0.592	0.613	0.629	0.632	0.634	0.636
	**CMN**	0.633	0.614	0.600	0.592	0.591	0.588
	**SAMN**	0.636	0.669	0.660	0.657	0.605	0.619
	**Without Rating Conversion**	0.654	0.643	0.633	0.628	0.628	0.627
	**Proposed Method**	**0.675**	**0.671**	**0.665**	**0.663**	**0.665**	**0.665**

**Table 6:** Comparison of the experimental results on ML-1M, Epinions, and Yelp datasets using the HR.

Datasets	Methods	HR@k					
		5	10	20	30	40	50
**ML-1M**	**SVD**	0.259	0.415	0.635	0.687	0.729	0.758
	**NMF**	0.239	0.387	0.619	0.671	0.715	0.749
	**CMN**	0.286	0.401	0.598	0.637	0.669	0.697
	**SAMN**	0.325	0.440	0.627	0.674	0.709	0.736
	**Without Rating Conversion**	0.290	0.404	0.628	0.670	0.707	0.734
	**Proposed Method**	**0.337**	**0.455**	**0.654**	**0.695**	**0.733**	**0.761**
**Epinions**	**SVD**	0.771	0.829	0.905	0.970	-	-
	**NMF**	0.748	0.797	0.876	0.948	-	-
	**CMN**	0.760	0.810	0.881	0.962	-	-
	**SAMN**	0.802	0.843	0.923	0.980	-	-
	**Without Rating Conversion**	0.774	0.823	0.919	0.979	-	-
	**Proposed Method**	**0.808**	**0.860**	**0.939**	**0.981**	-	-
**Yelp**	**SVD**	0.678	0.714	0.744	0.756	0.769	0.777
	**NMF**	0.665	0.704	0.736	0.749	0.760	0.769
	**CMN**	0.709	0.729	0.754	0.764	0.773	0.780
	**SAMN**	0.585	0.732	0.778	0.799	0.802	0.830
	**Without Rating Conversion**	0.729	0.729	0.755	0.769	0.775	0.793
	**Proposed Method**	**0.792**	**0.788**	**0.785**	**0.803**	**0.806**	**0.837**

The experimental results between the proposed method and the other methods using prediction accuracy metrics on three datasets are shown in Table 7. The proposed method achieves significantly higher performance than SVD, NMF, CMN, SAMN, and the proposed method without the rating conversion module when compared using precision and recall on all datasets. Moreover, the proposed method also obtains the lowest errors on the RMSE metric. Therefore, converting the neighbor’s preference range into the target user aspect and indicating the user similarity through the neighbors’ attentions can provide more accurate experimental results.

**Table 7:** Predicted accuracy experimental results.

Datasets	Metrics	SVD	NMF	CMN	SAMN	Without Rating Conversion	Proposed Method
**ML-1M**	**Precision**	0.798	0.787	0.766	0.788	0.781	0.811
	**Recall**	0.782	0.773	0.224	0.776	0.766	0.809
	**RMSE**	0.988	1.025	1.282	1.029	1.262	0.985
**Epinions**	**Precision**	0.769	0.751	0.719	0.772	0.728	0.789
	**Recall**	0.775	0.746	0.749	0.798	0.785	0.812
	**RMSE**	1.413	1.474	1.637	1.410	1.580	1.395
**Yelp**	**Precision**	0.779	0.766	0.840	0.849	0.846	0.866
	**Recall**	0.785	0.749	0.768	0.776	0.773	0.791
	**RMSE**	1.112	1.230	1.232	1.076	1.127	1.037.

To evaluate the effectiveness of the rating conversion module, we compare the proposed method with the proposed method without rating conversion. The experimental results are shown in Tables 5–7 and Fig 7. The proposed method achieves higher efficiency than the proposed method without the rating conversion at all evaluation metrics because the neighbors’ preference ranges are converted into the target user aspect. Moreover, the proposed method without the rating conversion can provide higher performance than the CMN but lower than the SAMN. Therefore, converting the neighbors’ ratings into the target user’s perspective view can make the prediction more accurate than not converting the neighbors’ ratings.

In additional, we compare the proposed method with latent factor models. The experimental results have shown in Tables 5–7. From the results show that the proposed method is perform better than the SVD and NMF, which are the latent factor model in both ranking-based evaluation and prediction accuracy.

*3.1.4.2 All neighbors*. From selecting *N* neighbors as neighbors, the 30 neighbors obtain the highest performance. Therefore, the result of the 30 neighbors is used to compare with all neighbors in this subsection. Table 8 show the evaluation results, which compare the nDCG results of the 30 neighbors (*N* = 30) and all neighbors.

**Table 8:** The experimental results of using *N* neighbor and all neighbor.

		ML-1M		Epinions		Yelp	
	@k	N = 30	All	N = 30	All	N = 30	All
**nDCG**	**5**	**0.391**	0.362	0.749	**0.756**	**0.675**	0.627
	**10**	**0.506**	0.435	0.783	**0.790**	**0.671**	0.663
	**20**	**0.675**	0.609	0.775	**0.809**	**0.665**	0.645
	**30**	**0.712**	0.640	0.783	**0.825**	**0.663**	0.598
	**40**	**0.742**	0.665	-	-	**0.665**	0.549
	**50**	**0.761**	0.685	-	-	**0.665**	0.543
**HR**	**5**	**0.337**	0.315	0.808	**0.811**	**0.792**	0.773
	**10**	**0.455**	0.438	0.860	**0.863**	**0.788**	0.766
	**20**	**0.654**	0.652	0.939	**0.940**	**0.785**	0.735
	**30**	**0.695**	0.686	0.981	**0.986**	**0.803**	0.782
	**40**	**0.733**	0.712	-	-	**0.806**	0.783
	**50**	**0.761**	0.734	-	-	**0.837**	0.822
**Precision**		**0.811**	0.787	0.789	**0.793**	**0.866**	0.852
**Recall**		**0.809**	0.803	0.812	**0.815**	**0.791**	0.784
**RMSE**		**0.985**	1.054	1.395	**1.341**	**1.037**	1.073

**Fig 8:**
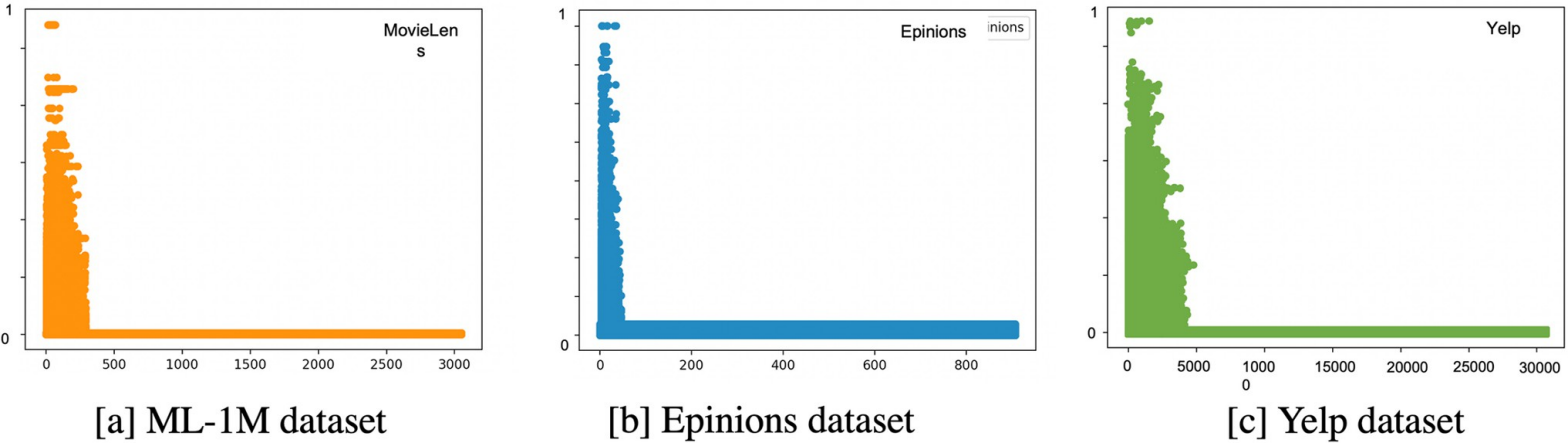
Similarity values between the neighbors and the target user of three datasets. [a] ML-1M dataset. [b] Epinions dataset. [c] Yelp dataset.

The results from ML-1M and Yelp dataset with using 30 neighbors obtain higher than using all neighbors in all evaluation metrics. While the Epinions dataset with all neighbors obtain the higher than using 30 neighbors. From Fig 8[a] and 8[c], it can be seen that almost similarity values of ML-1M and Yelp dataset are close to zero, which means almost neighbors are less similar to the target users in both dataset. In contrast, almost the similarity values in the Epinions dataset are close to one in Fig 8[b]. Therefore, the neighbors from the Epinions dataset have a high quality to use in recommendation than the ML-1M and Yelp datasets.

### 3.2 Syntatic datasets

Due to the real-world datasets being unable to control the data distribution, we generate the full rating matrix to compare the results using different rating distributions: the normal distribution, skewed right distribution, and skewed left distribution. The full rating matrix dataset is an ideal user–item dataset in CF, which each element in matrix is the rating of user row *i* th on item column *j*th. Besides these three distributions, we also generate the partial rating matrix, which is similar to the real-world dataset with normal rating distribution. To evaluate the performance of the syntetic datasets, the nDCG, HR, Precision, Recall, and RMSE metrics are used to evaluate the performance of the proposed method with the baselines.

#### 3.2.1 Data generation setting

**Fig 9:**
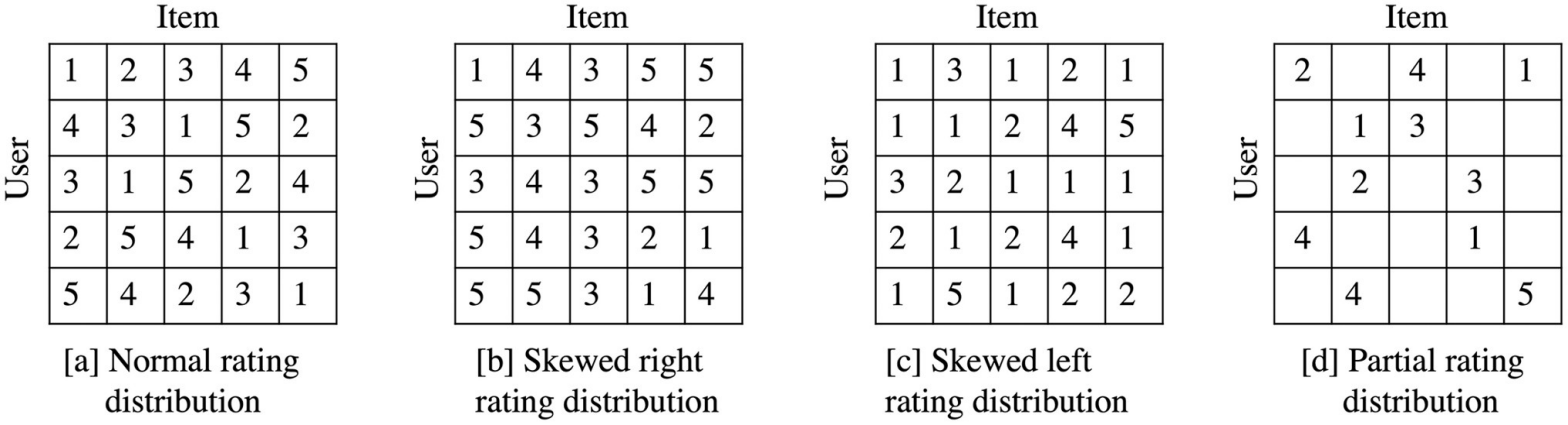
The example of synthetic datasets. [a] Normal rating distribution [b] Skewed right rating distribution. [c] Skewed left rating distribution. [d] Partial rating distribution.

In this work, the rating range of both full and partial rating matrices are set to range 1-5. In order to generate the datasets, there are four main parameters, which are the numbers of user, item, raters, and rating score. We desire to evaluate three types of distributions of the full rating matrix:

- *Normal rating distribution*: the dataset where the number of rating in each rating score is equal. Fig 9[a] is the example of the normal rating distribution, which each rating score has one rating.
- *Skewed right rating distribution*: the dataset where the number of high rating scores is more than the number of low rating scores. Fig 9[b] shows the example of skewed right rating distribution, which the number of score 3, 4 and 5 are more than score 1 and 2.
- *Skewed left rating distribution*: the dataset where the low rating score is more than the high rating score. The example of skewed right rating distribution shows in Fig 9[c]. The number of rating score 1 and 2 are more than score 3, 4 and 5.

Moreover, the size of each rating distribution depends on *k* of nDCG matrics to compare the effect of dataset size. In this work, we evaluate nDCG @*k* for *k* ∈ {5, 10, 20, 30, 40, 50}. Therefore, there are six datasets size in each distribution. The data in dataset is divided into ratio 80:20 for train:test sets. Suppose *k* = 5, the minimum number for user–item interaction that can evaluate using *k* = 5 is twenty five interaction. Therefore, we use the number of user, item, rater and total number of rating score equal to twenty five.

We simulate the normal distribution partial rating dataset, which more similar to the real-world dataset. The partial dataset is the dataset that the users have rated some items and has more additional rating data than the full rating dataset. The example of partial rating dataset has show in Fig 9[d]. The partial rating matrix is generated by using 1000-by-1000 rating matrix and fifty raters per items.

Table 9 shows the parameter setting of all synthetic dataset. The dataset gen1 to gen6 are the normal data distribution with different size. The datasets gen7 to gen12 are the skewed right data distribution and datasets gen13 to gen18 are the skewed left data distribution. The dataset gen19 is the partial dataset that has two hundred and fifty raters similar to the gen6. However, gen19 has more rating patterns than the gen6 dataset because the target user does not need to rate all items in the dataset.

**Table 9:** The parameters of simulating data.

dataset name	*k*	#user	#item	#rater	#1	#2	#3	#4	#5
**gen1**	5	25	25	25	5	5	5	5	5
**gen2**	10	50	50	50	10	10	10	10	10
**gen3**	20	100	100	100	20	20	20	20	20
**gen4**	30	150	150	150	30	30	30	30	30
**gen5**	40	200	200	200	40	40	40	40	40
**gen6**	50	250	250	250	50	50	50	50	50
**gen7**	5	25	25	25	2	3	5	7	8
**gen8**	10	50	50	50	3	5	10	15	17
**gen9**	20	100	100	100	5	10	20	30	35
**gen10**	30	150	150	150	10	15	30	45	50
**gen11**	40	200	200	200	10	20	40	60	70
**gen12**	50	250	250	250	15	20	50	80	85
**gen13**	5	25	25	25	8	7	5	3	2
**gen14**	10	50	50	50	17	15	10	5	3
**gen15**	20	100	100	100	35	30	20	10	5
**gen16**	30	150	150	150	50	45	30	15	10
**gen17**	40	200	200	200	70	60	40	20	10
**gen18**	50	250	250	250	85	80	50	20	15
**gen19**	50	1,000	1,000	250	50	50	50	50	50

#### 3.2.2 Experimental results

In this work, nineteen datasets are evaluated to study the three different data distributions and different sizes of the datasets. Table 10 shows the experimental results of all datasets by using nDCG. From Table 10, the proposed method obtained the highest nDCG results when compared with the baselines in terms of the ranking list.

**Table 10:** The nDCG experimental results of synthetic data.

dataset name	*k*	SVD	NMF	CMN	SAMN	Proposed method
**gen1**	5	0.716	0.757	0.758	0.748	**0.758**
**gen2**	10	0.695	0.681	0.695	0.695	**0.696**
**gen3**	20	0.623	0.640	0.640	0.638	**0.642**
**gen4**	30	0.613	0.611	0.615	0.614	**0.615**
**gen5**	40	0.581	0.589	0.595	0.594	**0.601**
**gen6**	50	0.578	0.578	0.579	0.578	**0.584**
**gen7**	5	0.779	0.794	0.816	0.814	**0.816**
**gen8**	10	0.742	0.750	0.751	0.743	**0.752**
**gen9**	20	0.705	0.695	0.712	0.711	**0.712**
**gen10**	30	0.694	0.684	0.695	0.694	**0.696**
**gen11**	40	0.677	0.679	0.680	0.680	**0.691**
**gen12**	50	0.682	0.682	0.687	0.688	**0.689**
**gen13**	5	0.745	0.742	0.746	0.746	**0.748**
**gen14**	10	0.663	0.688	0.693	0.695	**0.698**
**gen15**	20	0.632	0.630	0.643	0.647	**0.649**
**gen16**	30	0.589	0.585	0.583	0.588	**0.590**
**gen17**	40	0.578	0.580	0.586	0.589	**0.591**
**gen18**	50	0.558	0.553	0.566	0.567	**0.570**
**gen19**	50	0.674	0.570	0.564	0.573	**0.689**

In terms of data distribution, the experimental results show that the skewed right rating distribution dataset obtained the highest nDCG results compared to the normal rating distribution and the skewed left rating distribution. Since most rating scores in the skewed right data distribution are high rating scores, these datasets have many positive preferences. In contrast, the skewed left data distribution has a lot of negative preferences. Usually, making recommendations is more about suggesting things users like than suggesting things users do not. Therefore, using positive preference is more effective in recommendation than using negative preference. The normal rating distribution datasets have an equal number for each rating score, which means a number of positive preferences equal negative preferences. Thus, the normal rating distribution datasets obtained the lower nDCG result than the skewed right rating distribution dataset and higher nDCG results than the skewed left rating distribution dataset.

Regarding size comparison, the small dataset obtains higher nDCG than the large dataset because simulated datasets depend on *k*. It means the small dataset is less *k*, the large set is large *k*. Therefore, using a small number of *k* has a greater chance of ranking correctly than the large *k*.

The full normal distribution rating matrix, gen6, is the maximum number of raters used to evaluate in this work. Therefore, we created gen19 in a normal distribution with the number of raters equal to gen6. The dataset gen19 obtained a higher ranking quality than gen6. Because the partial rating dataset has more different patterns of interactions than gen6, which is gen19 has additional data in learning. Therefore, gen19 can predict the recommended item better than gen6.

The HR metrics use the list of the rating prediction in test set to evaluate how many ratings in *k*-sized list of ranked items are matched with the actual rating ranked items. Due to the number of interactions in simulated datasets are generated by *k*, the HR ranked items results of all simulated datasets is equal to *k*. It means all of the predicted ratings are used in HR evaluation. Therefore, the HR results of all simulated datasets is one.

The accuracy of experimental results using Precision, Recall, and RMSE are shown in Table 11. The experimental results of the proposed method obtain the highest prediction results when compare with the two latent factor models and two current NCF with friends.

**Table 11:** The accuracy experimental results of synthetic data.

Dataset name	Metrics	SVD	NMF	CMN	SAMN	Proposed method
**gen1**	**Precision**	0.508	0.585	0.391	0.391	**0.547**
	**Recall**	0.500	0.563	0.620	0.623	**0.625**
	**RMSE**	1.489	1.527	1.709	2.439	**1.220**
**gen2**	**Precision**	0.530	0.496	0.485	0.385	**0.539**
	**Recall**	0.502	0.453	0.388	0.619	**0.621**
	**RMSE**	1.438	1.686	1.729	2.403	**1.202**
**gen3**	**Precision**	0.492	0.512	0.357	0.357	**0.499**
	**Recall**	0.495	0.460	0.590	0.592	**0.597**
	**RMSE**	1.512	1.540	1.746	1.711	**0.873**
**gen4**	**Precision**	0.507	0.513	0.520	0.353	**0.525**
	**Recall**	0.480	0.467	0.516	0.594	**0.595**
	**RMSE**	1.509	1.487	1.434	1.638	**0.838**
**gen5**	**Precision**	0.506	0.510	0.518	0.354	**0.530**
	**Recall**	0.490	0.457	0.498	0.591	**0.594**
	**RMSE**	1.521	1.449	1.133	1.440	**1.031**
**gen6**	**Precision**	0.520	0.512	0.470	0.512	**0.517**
	**Recall**	0.495	0.482	0.392	0.521	**0.595**
	**RMSE**	1.540	1.446	1.350	1.380	**0.854**
**gen7**	**Precision**	0.640	0.623	0.586	0.586	**0.733**
	**Recall**	0.500	0.547	0.762	0.763	**0.766**
	**RMSE**	1.428	1.603	1.387	1.374	**1.372**
**gen8**	**Precision**	0.727	0.737	0.696	0.716	**0.857**
	**Recall**	0.549	0.571	0.822	0.825	**0.828**
	**RMSE**	1.230	1.288	1.237	1.726	**1.208**
**gen9**	**Precision**	0.732	0.727	0.709	0.709	**0.887**
	**Recall**	0.570	0.603	0.837	0.840	**0.842**
	**RMSE**	1.207	1.241	1.582	1.522	**1.184**
**gen10**	**Precision**	0.723	0.725	0.730	0.700	**0.897**
	**Recall**	0.500	0.404	0.511	0.527	**0.528**
	**RMSE**	1.251	1.238	1.695	1.734	**1.173**
**gen11**	**Precision**	0.745	0.741	0.723	0.753	**0.904**
	**Recall**	0.592	0.524	0.530	0.500	**0.549**
	**RMSE**	1.227	1.201	1.260	1.220	**1.143**
**gen12**	**Precision**	0.760	0.763	0.757	0.754	**0.868**
	**Recall**	0.608	0.640	0.705	0.841	**0.850**
	**RMSE**	1.227	1.176	1.212	1.359	**1.092**
**gen13**	**Precision**	0.493	0.440	0.453	0.493	**0.502**
	**Recall**	0.500	0.484	0.591	0.571	**0.608**
	**RMSE**	1.241	1.528	1.217	1.218	**1.214**
**gen14**	**Precision**	0.495	0.538	0.534	0.534	**0.610**
	**Recall**	0.504	0.580	0.566	0.566	**0.586**
	**RMSE**	1.247	1.358	1.238	1.238	**1.217**
**gen15**	**Precision**	0.538	0.541	0.544	0.564	**0.616**
	**Recall**	0.559	0.580	0.552	0.572	**0.593**
	**RMSE**	1.193	1.250	1.276	1.271	**1.172**
**gen16**	**Precision**	0.544	0.531	0.534	0.564	**0.556**
	**Recall**	0.502	0.565	0.563	0.566	**0.576**
	**RMSE**	1.262	1.268	1.258	1.252	**1.179**
**gen17**	**Precision**	0.534	0.543	0.553	0.524	**0.636**
	**Recall**	0.534	0.571	0.645	0.652	**0.708**
	**RMSE**	1.233	1.201	1.677	1.684	**1.212**
**gen18**	**Precision**	0.552	0.539	0.514	0.534	**0.660**
	**Recall**	0.530	0.579	0.538	0.549	**0.619**
	**RMSE**	1.224	1.181	1.264	1.264	**1.128**
**gen19**	**Precision**	0.517	0.505	0.357	0.537	**0.535**
	**Recall**	0.531	0.525	0.597	0.597	**0.896**
	**RMSE**	1.212	1.269	1.416	1.434	**1.201**

## 4 Discussions

Because the concept of neighbors is applied to a NN, issues of similarities between the neighbors and the target user and rating conversion need to be considered. Therefore, we proposed the similarity module and the rating conversion module, which can deal with both issues.

### 4.1 Performance of similarity module

In the CF technique, the prediction Eq (4) is a weighted average on the neighbors’ ratings where the similarity levels between the neighbor and the target user are used as a weight. The neighbors’ rating scores are provided by neighbors who are similar to the target user. The similarity shows how much neighbors and the target user are similar. The current NCFs with friends compute neighbor’s influence and include neighbor’s influence with the user’s representation to predict the target user’s rating score. Therefore, both current NCFs with friends do not use the neighbors’ ratings directly but combine these neighbors’ ratings with the similarity level between the neighbor and the target user directly as the CF technique’s rating prediction equation. In comparison, the proposed method uses the attention of neighbors to compute the similarity levels in the fourth step. Similarities between the neighbors and the target user were used as a weight for computing weighted average in the rating prediction step. In this step, the proposed method’s rating prediction imitates the CF technique’s rating prediction equation, which produces the neighbors’ ratings and the similarity levels between the neighbor and the target user. Therefore, it makes the proposed method more accurate than the current NCFs with friends.

### 4.2 Performance of rating conversion module

According to the CF concept, the neighbors’ preferences are used to predict the target user’s ratings. However, different user has a different rating range, although they have similar preferences. This is one of the issues in the CF approach that is not often discussed. Therefore, we would like to evaluate the real efficiency of our rating conversion module by remove the converting neighbor in to the target user aspect. The experimental results in Tables 5–7 and Fig 7 show that the proposed method obtained the highest efficiency because the neighbors’ preferences are converted into the target user aspect. Both current NCFs with friends predict the ratings using users’ representations, items’ representations, and the neighbor’s influences, which did not concern the rating conversion issue. The proposed method concerns and solves the rating conversion issue in the rating conversion module. In this module, the neighbors’ rating ranges are converted into the target user aspect by element-wise multiplication between the target user’s LCU and the neighbors’ representations. Afterward, the converted neighbors’ representation was used to compute the neighbors’ rating in the target user’s perspective view.

### 4.3 Neighbor selection

The traditional CF both the neighbor’s rating and the similarity between the neighbor and the target user are used to perform the rating prediction. Therefore, the number of neighbors is essential in CF research. Due to using all raters as a neighbors may make a incorrect prediction because the low-quality neighbors. In this work, the proposed method is evaluated using *N* neighbors and all neighbors as neighbors. The experimental results in Table 8 shown that using all neighbors to predict the rating obtain a lower performance than using *N* neighbors in ML-1M and Yelp dataset. Due to most of neighbors in both datasets have the similarity values close to the zero, which means there are many low-quality neighbors. Therefore, using all neighbors on ML-1M and Yelp datasets perform lower accuracy than using *N* neighbors in prediction.

In contrast, the results of the Epinions dataset using all neighbors are obtained higher than using *N* neighbors. From Fig 8, almost all the similarity values are close to one. Most of the neighbors in the Epinions dataset are high-quality neighbors. Thus, using all neighbors can improve the prediction results. To summarize, two of three in real-world datasets use *N* neighbors better performance than using all neighbors.

### 4.4 Data simulation

There are two types of synthetic datasets in this work: full rating dataset and partial rating dataset. When comparing the full rating dataset and the partial rating dataset in a normal distribution, the partial rating dataset performs better than the full rating dataset. The reason is the partial rating dataset has more rating patterns than the full rating dataset. For example, there are three items in the dataset. In the case of a full rating dataset with normal distribution, six patterns of rating can occur in each user. While the partial rating dataset with a normal distribution, twenty-four rating patterns can occur because the users do not need to rate all items in the dataset. Therefore, the model can learn many rating patterns in the partial rating dataset, which make the partial rating dataset outperforms the full rating dataset.

In the full rating dataset, the rating distribution and the dataset sizes are considered. There are three types of rating distribution: the normal rating distribution, skewed right rating distribution, and skewed left rating distribution. Table 10 shown that the skewed right rating distribution performs better than the other two distributions. Due to the nature of the recommendation, the positive preferences of users are used to performing the suggestion more than negative preferences.

**Fig 10:**
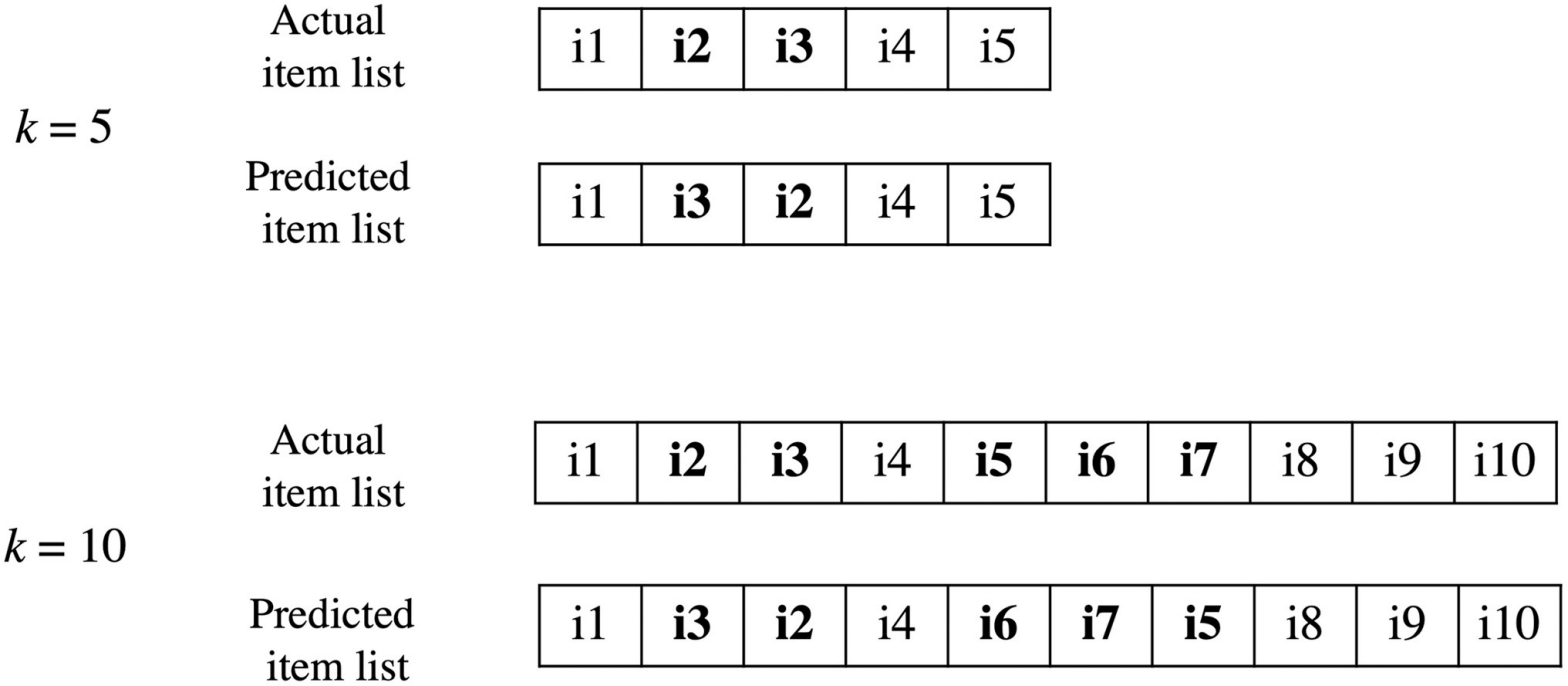
The example of comparing *k* ranking.

Under the distribution condition, we simulate the size of rating matrix depending on *k* of nDCG. The results shown that the small dataset obtained higher performance than the large dataset. Because the small dataset uses the less *k* to make ranking and the large dataset uses the large *k* to create the ranking, which is the less *k* has more chance to rank the predicted rating correctly than the large *k*. For example Fig 10, it can be seen that the ranking *k* = 5, there is higher probability to raking correctly than *k* = 10.

When comparing all evaluation results of our proposed method with other methods, the proposed method outperforms all the current NCF with friends, CMN and SAMN, and the latent factor models, SVD and NMF. In the case of the current NCF with friends, the CMN and SAMN perform only the similarity between the neighbor and the target user. In comparison, the proposed method computes the similarity between the neighbor and the target user and applies the converted neighbors’ ratings in the prediction process, as discussed in Sections 4.1 and 4.2. In the case of the latent factor models, the reasons are the same as using real-world datasets, which are discussed in Section 4.5.

### 4.5 Comparing method with latent factor model

In order to evaluate the proposed method with the latent factor model, we use SVD and NMF as baselines of our experiment and evaluate on a real-world and simulated dataset. The experimental results show that the proposed method performs better than both SVD and NMF. According to previous works, NCF and current NCF with friends perform better than MF, consequently. In this work, we also prove that our proposed method is better than the latent factor model. Because using NN make the model can capture the non-linear data distribution and more latent dimension than the latent factor model.

## 5 Conclusions

We proposed strategies considering the similarity and the rating conversion of neighbors on NCF, which applies neighbors’ concept of the CF to a NN. Besides, the proposed method can also deal with the similarity between the neighbors and the target user and the rating conversion issues. Because different users have different preference ranges, which makes inaccurate predictions, the rating conversion should be considered. The similarity between the neighbors and the target user is one crucial factor that is used to obtain the rating prediction in the CF technique.

The outcome of the proposed method is the target users’ rating prediction, which is obtained using a weighted average on the neighbors’ ratings where the similarities between the neighbors and the target user are used as a weight according to the rating prediction of the CF technique. These similarities are obtained using the neighbor’s attention, which is a dot product between the target user and the neighbor representation. The neighbors’ ratings are computed using the MF concept between the item’s characteristics and the converted neighbors’ representations.

In the experiment, we evaluate our proposed method on the real-world datasets and the simulation datasets and compare with existing methods and the latent factor models. The results show that the proposed method outperform all baselines using ranking-based evaluation and prediction accuracy metrics. To evaluate the efficiency of the rating conversion in the real-world dataset, we compare the proposed method with the proposed method without the rating conversion in the real-world dataset. The results show that the proposed method performs higher efficiency than the proposed method without the rating conversion. In order to know the effectiveness of the number of neighbors, we divided using neighbor into two types: the *N* neighbors and all neighbors. The experimental result shows that using *N* neighbors obtain a better performance than using all neighbors in a real-world dataset. Moreover, we evaluate the effectiveness of using different rating distribution and datasets sizes. In terms of rating distribution, the skewed right rating distribution performs the best performance in synthetic datasets. In terms of dataset size, the small datasets perform better than the large datasets.

From Fig 5, because of the limitation of NN implementation, the number of neighbors must be equal for every target user. Therefore, we need to fix the maximum number of neighbors to learn the model. However, in reality, different target users do not need to have the same number of neighbors. In the future, if a NN model can be implemented using the actual number of neighbors, it may improve the performance and make the model more realistic.

## References

[1] Isinkaye F.O., Folajimi Y.O., Ojokoh B.A. Recommendation systems: Principles, methods and evaluation. Egyptian Informatics Journal. 2015:261–273. doi: 10.1016/j.eij.2015.06.005

[2] Robin B., Felfernig A., and Göker M. H. Recommender Systems: An Overview. Ai Magazine. 2021:13–18.

[3] Kim B., Li Q., Park C. S., Kim S. Q., and Kim J. Y. A new approach for combining content-based and collaborative filters. J. Intell. Inf. Syst., 2006:79–91, 2006. doi: 10.1007/s10844-006-8771-2

[4] Nilashi M., Bagherifard K., Ibrahim O., Alizadeh H., Nojeem L. A., and Roozegar N. (2013). Collaborative Filtering Recommender Systems. Research Journal of Applied Sciences, Engineering and Technology. 2013;4:4168–4182. doi: 10.19026/rjaset.5.4644

[5] Burke R. Hybrid Recommender Systems: Survey and Experiments. User Modeling and User-Adapted Interaction 12. 2002;11: 331–370. doi: 10.1023/A:1021240730564

[6] Shah S. Introduction to Matrix Factorization for Recommender Systems. ResearchGate. 2018;12.

[7] Bokde D., Girase S., and Mukhopadhyay D. Matrix Factorization Model in Collaborative Filtering Algorithms: A Survey. Procedia Computer Science. 2015: 136–146. doi: 10.1016/j.procs.2015.04.237

[8] Koren Y., Bell R., and Volinsky C. Matrix Factorization Techniques for Recommender Systems. IEEE Computer Society. 2009;8:30–37. doi: 10.1109/MC.2009.263

[9] M. Richardson. Principal Component Analysis. 2009.

[10] B. Kirk. Singular Value Decomposition Tutorial. 2005. Available from: https://datajobs.com/data-science-repo/SVD-Tutorial-[Kirk-Baker].pdf

[11] Blei D. M., Ng A. Y., and Jordan M. I. Latent Dirichlet Allocation. Journal of Machine Learning Research 3, 2003:993–1022.

[12] Rahmani H.A., Aliannejadi M., Ahmadian S., Baratchi M., Afsharchi M., Crestani F. LGLMF: Local Geographical Based Logistic Matrix Factorization Model for POI Recommendation. Springer: Information Retrieval Technology, 2009:pp 66-78.

[13] WU D., He Q., Luo X., Shang M., He Y. and Wang G. A Posterior-neighborhood-regularized Latent Factor Model for Highly Accurate Web Service QoS Prediction. IEEE Transactions on Services Computing. 2019;12:1–12.

[14] K. L. Du, and M.N.S. Swamy. Recurrent Neural Networks. 2014:12.

[15] S. Albawi, T. A. Mohammed and S. Al-Zawi. Understanding of a convolutional neural network. 2017 International Conference on Engineering and Technology (ICET). 2017:1-6.

[16] Hochreiter S. and Schmidhuber J. Long Short-Term Memory. in Neural Computation. 1997;11:1735–1780. doi: 10.1162/neco.1997.9.8.1735 9377276

[17] F. A. Gers, J. Schmidhuber and F. Cummins. Learning to forget: continual prediction with LSTM. 1999 Ninth International Conference on Artificial Neural Networks ICANN 99. 1999:850-855.

[18] Gers F. A. and Schmidhuber E. LSTM recurrent networks learn simple context-free and context-sensitive languages. IEEE Transactions on Neural Networks. 2001: 1333–1340. doi: 10.1109/72.963769 18249962

[19] C. Zhao, X. Shi, M. Sheng, and Y. Fang. A Clustering-Based Collaborative Filtering Recommendation Algorithm via Deep Learning User Side Information. International Conference on Web Information Systems Engineering (WISE 2020). 2020:331-342.

[20] X. He, L. Liao, H. Zhang, L. Nie, X. Hu, and T. Chua. Neural Collaborative Filtering. ACM 26th International Conference on World Wide Web (WWW’17). 2017: 173-182.

[21] X. He X. Du X. Wang, F. Tian, J. Tang, and T. Chua. Outer Product-based Neural Collaborative Filtering. the Twenty-Seventh International Joint Conference on Artificial Intelligence (IJCAI-18). 2019:2227-2233.

[22] Y. Mao, X. Shi, M. Shang, and Y. Zhang. TCR: Temporal-CNN for Reviews Based Recommendation System. ACM 2nd International Conference on Deep Learning Technologies (ICDLT’18). 2018: 71-75.

[23] Bai T., Wen J., Zhang J., and Zhao W. X. A Neural Collaborative Filtering Model with Interaction-based Neighborhood. Information and Knowledge Management (CIKM ‘17). 2017;11: 1979–1982.

[24] W. Xi, L. Huang, C. Wang, Y. Zheng, and J. Lai. BPAM: Recommendation Based on BP Neural Network with Attention Mechanism (IJCAI-19). the Twenty-Eighth International Joint Conference on Artificial Intelligence. 2019:3905-3911.

[25] T. Ebisu, B. Shen, and Y. Fang. Collaborative Memory Network for Recommendation Systems. The 41st International ACM SIGIR Conference on Research Development in Information Retrieval (SIGIR’18). June 2018:6:515–524.

[26] C. Chen, M. Zhang, Y. Liu, and S. Ma. Social Attentional Memory Network: Modeling Aspect- and Friend-Level Differences in Recommendation. Proceedings of the Twelfth ACM International Conference on Web Search and Data Mining (WSDM’19), 2019;1:177–185.

[27] T. Mikolov, I. Sutskever, K. Chen, G. Corrado, and J. Dean. Distributed representations of words and phrases and their compositionality. Proceedings of the 26th International Conference on Neural Information Processing Systems (NIPS’13). 2013;12: 3111–3119.

[28] A. Joulin, E. Grave, P. Bojanowski, T. Mikolov. Bag of Tricks for Efficient Text Classification. 15th Conference of the European Chapter of the Association for Computational Linguistics. 2017:427-431.

[29] W. Chalermpornpong, S. Maneeroj, A. Takasu. Rating Pattern Formation for Better Recommendation. 24th International Workshop on Database and Expert Systems Applications. 2013:146-151.

[30] C. Qiao, B. Huang, G. Niu, D. Li, D. Dong, W. He, et al. A New Method of Region Embedding for Text Classification. 6th International Conference on Learning Representations (ICLR2018). 2018: 1-12.

[31] grouplens (2003). MovieLens 1M Dataset. https://grouplens.org/datasets/movielens/1m/

[32] Paolo Massa (2003). Epinions ratings data. http://www.trustlet.org/epinions.html

[33] Luís Fred (2018). Yelp Reviews CSV. Kaggle. 1. https://www.kaggle.com/luisfredgs/yelp-reviews-csv

[34] D. P. Kingma and J. L. Ba. Adam: A Method for Stochastic Optimization. 3rd International Conference on Learning Representations (ICLR). 2015;5:7-9.

[35] S. Ruder. An overview of gradient descent optimization algorithms. ArXiv abs/1609.04747, 2016. Available from: https://arxiv.org/abs/1609.04747

[36] E. M. Dogo, O. J. Afolabi, N. I. Nwulu, B. Twala and C. O. Aigbavboa. A Comparative Analysis of Gradient Descent-Based Optimization Algorithms on Convolutional Neural Networks. 2018 International Conference on Computational Techniques, Electronics and Mechanical Systems (CTEMS). 2018:92-99.

